# Coffee Compounds Protection Against Lipotoxicity Is Associated with Lipid Droplet Formation and Antioxidant Response in Primary Rat Hepatocytes

**DOI:** 10.3390/antiox14020175

**Published:** 2025-01-31

**Authors:** Johanna C. Arroyave-Ospina, Magnolia Martínez, Manon Buist-Homan, Victoria Palasantzas, Marco Arrese, Han Moshage

**Affiliations:** 1 Department of Fisiología y Bioquímica and Grupo de Gastrohepatología, Facultad de Medicina Universidad de Antioquia, Medellín 050010, Colombia; 2 Department of Gastroenterology and Hepatology, University Med ical Center of Groningen, University of Groningen, P.O. Box 30.001, 9713 GZ Groningen, The Netherlands; l.m.martinez.aguilar@umcg.nl (M.M.); m.buist-homan@umcg.nl (M.B.-H.); 3 Department of Genetics and Department of Pediatrics, University Medical Center of Groningen, University of Groningen, P.O. Box 30.001, 9713 GZ Groningen, The Netherlands; v.e.j.palasantzas@umcg.nl; 4 Department of Gastroenterology, Pontificia Universidad Católica de Chile, Santiago 7820436, Chile; marrese@uc.cl

**Keywords:** caffeine (CF), chlorogenic acid (CGA), lipotoxicity, oxidative stress, antioxidant response, lipid droplets (LDs), Stearoyl-CoA Desaturase 1 (SCD-1)

## Abstract

Metabolic dysfunction associated with steatotic liver disease (MASLD) is the result of disturbed lipid metabolism. In MASLD, the accumulation of free fatty acids (FFAs) in hepatocytes causes lipotoxicity mediated by oxidative stress. Coffee compounds are known for their beneficial effects in MASLD; however, the mechanisms still need to be further explored. The aim of this study was to elucidate the protective mechanisms of coffee compounds against palmitate-induced lipotoxicity in primary hepatocytes. Methods: Primary hepatocytes were isolated from male Wistar rats and treated with palmitate (1 mmol/L) in combination with caffeine (CF: 1 mmol/L) or chlorogenic acid (CGA: 5 µmol/L). Mitochondrial ROS production, palmitate-induced necrosis, antioxidant response, ER stress markers and lipid droplet (LD) formation were assessed. Monoacylglycerols 2-SG (2-Stearolylglycerol), 2-OG (2-Oleoylglycerol) and SCD-1 (Stearoyl-CoA Desaturase 1) inhibitors were used to modulate LD formation. LD formation in steatotic Zucker rat hepatocytes was also investigated. Results: CF and CGA prevented palmitate-induced cell death and reduced ROS production. CF and CGA induced the antioxidant response, especially HO-1 expression, but had no significant effect on ER stress markers. CF and CGA increased LD formation in palmitate-treated cells. This effect was significantly reduced by 2-SG and SCD-1 inhibitors but enhanced by 2-OG. Lipid droplets were associated with lower palmitate toxicity and reduced ROS production. Conclusions: CF and CGA protect hepatocytes from lipotoxicity via modulation of the antioxidant response and enhance lipid droplet formation via an SCD-1-dependent mechanism. Oxidative stress-related toxicity in hepatocytes can be prevented by enhancing LD formation.

## 1. Introduction

Non-alcoholic fatty liver disease (NAFLD) has recently been renamed metabolic dysfunction-associated steatotic liver disease (MASLD) based on modified criteria to better reflect the pathophysiology of the disease and improve risk prediction and outcome evaluation [1,2]. These diagnostic criteria include the confirmation of hepatic steatosis (fat accumulation > 5%) and the presence of one of the following risk factors: type II diabetes, overweight or obesity, or normal weight in combination with alterations of at least two metabolic risk parameters [3]. MASLD has a high prevalence and poses serious health threats, yet there is currently no standardized pharmacological therapy approved, although several drugs are under clinical investigation [4]. Therefore, there is an urgent need for novel therapeutic targets.

The pathophysiology of MASLD involves alterations of lipid metabolism and oxidative stress as a consequence of free fatty acid (FFA) overload in the liver. This causes hepatocyte lipotoxicity, triggering organelle dysfunction, inflammation and cell death [5]. Current evidence supports the notion that saturated FFAs are the main drivers of lipotoxicity [6], whereas lipid accumulation in the form of triglycerides is non-toxic and, in fact, constitutes a protective response against FFA-induced toxicity [7]. Triglycerides are stored in hepatocytes in the form of lipid droplets (LDs). It is becoming increasingly clear that LDs are not passive storage vehicles for triglycerides but play an active role in cellular homeostasis as key regulators of metabolism and it has been proposed that LDs modulate ROS production and oxidative stress [8]. Recent findings also suggest that LDs play a role in the modulation of lipotoxicity, especially in the protection against saturated FFA toxicity. LDs regulate several signaling pathways as well as gene expression and LDs may also be involved in the sequestration of toxic lipid species, protecting cells against lipotoxicity and oxidative stress [9,10]. In this regard, the functional role and mechanisms of LDs in lipotoxicity still need to be elucidated, as well as its potential as a therapeutic target.

Substantial epidemiological evidence supports the notion that coffee consumption is associated with beneficial health effects by reducing the risk of metabolic diseases [11], including the development and progression of MASLD [12,13]. Coffee compounds have been proposed as potential therapeutic compounds for MASLD based on experimental evidence that demonstrates their hepatoprotective effects [14]. Coffee has also been proposed as one of the most relevant dietary sources of antioxidant compounds and it has been shown that coffee consumption improves oxidative stress markers and increases antioxidant capacity in healthy adults [15,16]. Coffee beverages contain numerous bioactive compounds [17]. Coffee is an important source of caffeine (CF: 1,3,7-Trimethylxanthine). Coffee is also considered the major dietary source of chlorogenic acids (CGAs), a group of polyphenolic (ester) compounds [16]. Both CF and CGA have antioxidant properties that modulate oxidative stress, prevent ROS production and reduce inflammation [18,19,20,21,22]. Coffee compounds also modulate lipid metabolism [23]. Previously, we demonstrated that CF prevented palmitate-induced lipid toxicity in primary rat hepatocytes [24]. CGAs have also been shown to attenuate in vitro palmitate-induced toxicity in hepatocytes by preventing ROS production and ER stress [25,26]. However, the mechanisms and molecular targets that mediate these protective effects in the context of MASLD and lipotoxicity still need to be elucidated.

The aim of the present study was to elucidate the protective mechanisms of the coffee compounds CF and CGA against palmitate-induced lipotoxicity in primary rat hepatocytes, with a particular emphasis on the role of lipid droplets in these effects.

## 2. Materials and Methods

### 2.1. Coffee Compounds and Stock Solutions

Caffeine (C0750 Sigma Aldrich, Saint Louis, MO, USA) and chlorogenic acid (C3878 Sigma Aldrich) were dissolved in deionized water to prepare stock solutions and then sterilized by filtration using nitrocellulose membrane (0.25 µm) (Figure S1). Glutathione monoethyl ester (353905 Sigma Aldrich) and N-acetyl-L-cysteine (A7250 Sigma Aldrich) were used as established antioxidant compounds (Figure S1) and dissolved according to the manufacturer’s instructions. Menadione sodium bisulfite (M2518 Sigma Aldrich) and diclofenac sodium salt (D6899 Sigma Aldrich) were used as positive controls for ROS production. Palmitate stock solution (20 mmol/L) was prepared using sodium palmitate (Sigma Aldrich P9767) and sodium oleate in an aqueous solution of bovine serum albumin (BSA fatty acid-free) as previously described [27]. Free fatty acid mix (FFA) was prepared by mixing oleate and palmitate solutions at a 2:1 ratio, obtaining a final concentration of 0.75 mmol/L (0.5 mmol/L: oleate and 0.25 mmol/L: palmitate). Saturated 2-SG (2-Stearolylglycerol 36484), monounsaturated 2-OG (2-Oleoylglycerol M2787, Sigma Aldrich) and Stearoyl-CoA Desaturase 1 inhibitor (SCD-1 inhibitor 569406, Sigma Aldrich) were dissolved according to the manufacturer’s instructions.

### 2.2. Animal Ethics Statements

Experiments were performed according to the Dutch law on the welfare of laboratory animals (The Animal Act 2011), with permission no. 16778-01-002 from the Committee for Care and Use of Laboratory Animals of the University of Groningen. Specified pathogen-free male Wistar rats (220–250 g) aged 5–8 weeks and Zucker rats (12–14 weeks) were purchased from Charles River Laboratories Inc (Wilmington, MA, USA). Animals were housed in polypropylene cages at room temperature (25 ± 2 °C) with standard bedding, a regular cycle (12 h light/12 h dark) and free access to a standard chow diet and water at the animal facility center of the University Medical Center of Groningen. During hepatocyte isolation, animals were first placed for 5 min in a chamber containing 5% isoflurane. For anesthesia, a combination of ketamine (100 mg/mL, 60 mg/kg body weight) and medetomidine hydrochloride (1 mg/mL, 0.5 mg/kg body weight) was used.

### 2.3. Primary Rat Hepatocyte Isolation and Culture Conditions

Primary rat hepatocytes were isolated from male, pathogen-free Wistar rats (180–250 g and Zucker rats (ZDF: Fa/Fa and lean: Fa/+) using the two-step collagenase perfusion method according to the protocol previously described [28]. Cell viability was measured by the trypan blue exclusion method and only cell isolations with a viability above 80% were used for experiments. Hepatocytes were cultured in William’s E medium (Invitrogen, Breda, The Netherlands) supplemented with 5% fetal bovine serum supplemented with 50 nmol/L dexamethasone (Sigma-Aldrich, Zwijndrecht, The Netherlands), 50 μg/mL gentamicin (Invitrogen, Breda, The Netherlands), 100 U/mL penicillin, 100 μg/mL streptomycin and 250 ng/mL fungizone (1% PSF, Lonza, Verviers, Belgium) at 37 °C in a 5% (*v*/*v*) CO_2_-containing atmosphere. Palmitate and oleate stock solutions were prepared using sodium palmitate (Sigma Aldrich P9767) or oleate in an aqueous solution of free fatty acid-free bovine serum albumin (FFA-free BSA) as previously described [29]. After 4 h of attachment, hepatocytes were treated overnight (16 h) with a free fatty acid mix at 750 µmol/L (250 µmol/L palmitate plus 500 µmol/L oleate) or with oleate alone (OA, 500 µmol/L) to induce lipid droplet formation. For experiments with the coffee compounds, based on previous reports [24,26], we tested a range of concentrations between 0.05 and 1.5 mmol/L for CF and CGA. Hepatocytes were pretreated with CF (1 mmol/L) or CGA (5 μmol/L) and then co-treated with a toxic concentration of palmitate (1 mmol/L) or menadione (50 μmol/L) to induce ROS production. GSH-MEE (5 mmol/L) and NAC (5 mmol/L) were used as controls for antioxidant effects. 2-SG and 2-OG were used as saturated and monounsaturated monoacylglycerol, respectively. Hepatocytes were treated in serum-free and dexamethasone-free medium, supplemented as described above.

### 2.4. Cell Death Assessment (SYTOX Green)

To determine cell death, SYTOX^®^ green nucleic acid stain (S7020 Invitrogen, Carlsbad, CA, USA) was used as previously reported. Hepatocytes were treated with palmitate (1 mmol/L) with or without test compounds for 24 h. SYTOX green solution (125 nmol/L) was added to the cells after the treatment for 15 min at 37 °C in a 5% (*v*/*v*) CO_2_-containing atmosphere. Necrotic cells with disrupted membrane integrity were stained green and visualized using a Leica DMI6000 fluorescence microscope (Leica, Amsterdam, The Netherlands) at 512–542 nm. SYTOX green-positive cells were quantified using ImageJ. Briefly, the total number of SYTOX-positive cells (green nuclei) was counted per image and three different images were analyzed per condition. To avoid bias because of high cell death percentage in some conditions, normalization was performed for each image by calculating cell area percentage (cell confluency), and the relative number of SYTOX-positive cells was calculated compared to the BSA control.

### 2.5. Caspase-3 Activity Assay

Caspase-3 enzyme activity was used to determine apoptosis and assayed as described previously [29]. Fluorescence was quantified using a spectrofluorometer using an excitation wavelength of 380 nm and an emission wavelength of 460 nm. The arbitrary fluorescence units were normalized to the control condition and expressed as fold induction vs. control (FIC) to avoid unwanted sources of variation.

### 2.6. Mitochondrial and Total ROS Measurement

Mitochondrial ROS production was evaluated using the MitoSOX^TM Red^ superoxide indicator (M36008 Molecular Probes Inc., Eugene, OR, USA). Cells were seeded on glass coverslips and treated for 2 h with test substances. Cells were subsequently washed with warm HBSS buffer and stained with 2.5 μmol/L MitoSOX^TM Red^ superoxide indicator for 15 min. Cells were then washed three times with buffer and mounted on cover glasses with DAPI antifade mounting medium (Vectashield, Vectorlabs Mowry Avenue, Newark, NJ, USA). Cells were visualized using a Leica DMI6000 fluorescence microscope (Leica, Amsterdam, The Netherlands) at 590 nm. Quantitative analysis of the fluorescent images was performed using ImageJ.

### 2.7. Lipid Droplet Staining and Distribution

To visualize lipid droplets, a BODIPY 493/503 probe (D3922 Molecular Probes, Inc Invitrogen. Bleiswijk, The Netherlands) was used according to the protocol previously described (Qiu and Simon et al. 2016). Co-staining with MitoTracker™ Red CMXROS (M7512 Molecular Probes, Inc Invitrogen. Bleiswijk, The Netherlands) was used to visualize mitochondria and determine the subcellular distribution of lipid droplets. Briefly, cells were seeded on glass coverslips and treated with test substances for 24 h. Cells were subsequently washed with warm HBSS buffer, stained with BODIPY solution (2 μmol/L) and Mitotracker (200 nmol/L) for 15–20 min, and then washed and fixed with 3.7% paraformaldehyde. Coverslips were mounted on cover glasses with DAPI antifade mounting medium (Vectashield). Images were visualized using a Leica DMI6000 fluorescence microscope (Leica, Amsterdam, The Netherlands) at 512–524 nm. Image analysis was performed using ImageJ.

### 2.8. Immunofluorescent Staining

For the fluorescent staining of MnSOD (manganese superoxide dismutase: SOD2) protein, cells were seeded on glass coverslips and treated for 8 h. After treatment, cells were washed with HBSS buffer, subsequently fixed with 3.7% paraformaldehyde solution for 10 min and then permeabilized with 0.1% Triton X-100 for 30 min at 37 °C. Blocking was performed by incubation with BSA/PBS 2% solution for 30 min followed by incubation with primary antibody (1:200) for 1 h at room temperature. Anti-MnSOD (Stressgen ADI-SOD-111) was used as the primary antibody and, for the detection of the secondary antibody, goat anti-rabbit conjugated with Alexa-fluor 488 (1:400) was used (Invitrogen). Cells were then washed three times with buffer, mounted on cover glasses with DAPI antifade mounting medium (Vectashield) and visualized using a Leica DMI6000 fluorescence microscope at 512–542 nm.

### 2.9. RNA Isolation and Quantitative RT-PCR (qRT-PCR)

For RNA analysis, cells were harvested 6 h after treatment. mRNA levels of the ER stress-related genes GRP78, CHOP and ATF4 were determined, as well as various antioxidant-response genes: heme-oxygenase-1 (HO-1), manganese superoxide dismutase (MnSOD or SOD2), superoxide dismutase 1 (SOD1) and glutathione peroxidase 1 (GPx1). First, cells were washed twice with cold buffer and total RNA was isolated using TRI-reagent (Sigma-Aldrich) according to the manufacturer’s instructions. Reverse transcription (RT) was performed using 2.5 μg of total RNA, 1X RT buffer (500 mmol/L Tris-HCl [pH 8.3]; 500 mmol/L KCl; 30 mmol/L MgCl_2_; 50 mmol/L DTT), 1 mmol/L deoxynucleotides triphosphate (dNTPs, Sigma-Aldrich), 10 ng/μL random nanomers (Sigma-Aldrich), 0.6 U/μL RNaseOUT™ (Invitrogen, Carlsbad, CA, USA) and 4 U/μL M-MLV reverse transcriptase (Invitrogen) at a final volume of 50 μL. The cDNA synthesis program was 25 °C/10 min, 37 °C/60 min and 95 °C/5 min. Complementary DNA (cDNA) was diluted 20× in nuclease-free water. Real-time qPCR was carried out in a StepOnePlus™ (96-well) PCR System (Applied Biosystems, Thermo Fisher, Wilmington, DE, USA) using TaqMan probes. For qPCR, 2× reaction buffer (dNTPs, HotGoldStar DNA polymerase, 5 mmol/L MgCl_2_) (Eurogentec, Seraing, Belgium), 5 μmol/L fluorogenic probe and 50 μmol/L of sense and antisense primers (Invitrogen) were used. mRNA levels were normalized to the 18S housekeeping gene and further normalized to the mean expression level of the control. Primer sequences are listed in Supplementary Table S1.

### 2.10. Western Blot Analysis

Total protein lysates were prepared using lysis buffer (50 mmol/L Tris-base, pH 7.4, 0.2% Triton X-100, 0.25% Na-deoxycholate, 150 mmol/L NaCl, 1 mmol/EDTA) supplemented with protease inhibitors. Cell lysates were resolved on Mini-PROTEAN^®^ TGX Stain-Free™ Precast Gels (BioRad, Oxford, UK). Semi-dry-blotting was performed using a Trans-Blot Turbo Midi Nitrocellulose Membrane with Trans-Blot Turbo System Transfer (BioRad). Ponceau S 0.1% *w/v* (Sigma-Aldrich) staining was used to confirm protein transfer. Specific antibodies were used to detect HO-1: Anti-HO-1 (Stressgen SPA-896), MnSOD (SOD2): Anti-MnSOD (Stressgen ADI-SOD-111), Nrf2 protein: Anti-Nrf2 (Abcam 31163) and GRP78: Anti-GRP78 (Cell Signaling #3177). Anti-GAPDH (Calbiochem #CB1001) or anti-α-tubulin (Sigma Aldrich T9026) was used as a loading control. Appropriate anti-rabbit or anti-mouse peroxidase-conjugated secondary antibodies were used. Blots were analyzed with a ChemiDoc XRS system (Bio-Rad). Protein band intensities were quantified by ImageLab 6.1 software (BioRad).

### 2.11. Statistical Analysis

All the experiments were performed using hepatocytes from at least three independent isolations (*n* = 3) and with two replicates for each experimental condition. Normality tests were performed using Kolmogorov–Smirnov or Shapiro–Wilk test. The statistical significance of differences between the means of the experimental groups was evaluated using one-way and two-way analysis of variance (ANOVA) and the t-Student test as a post-test. The results are presented as the mean ± standard deviation (mean ± SD) with ns, *p* > 0.05, *p* ≤ 0.05, *p* ≤ 0.01, *p* ≤ 0.001 and *p* ≤ 0.0001. Analyses were performed using GraphPad Prism software 9 (Version 9.4.1, GraphPad Software Inc., San Diego, CA, USA).

## 3. Results

### 3.1. Coffee Compounds and Antioxidant Compounds Prevent Palmitate-Induced Lipotoxicity

**Figure 1:**
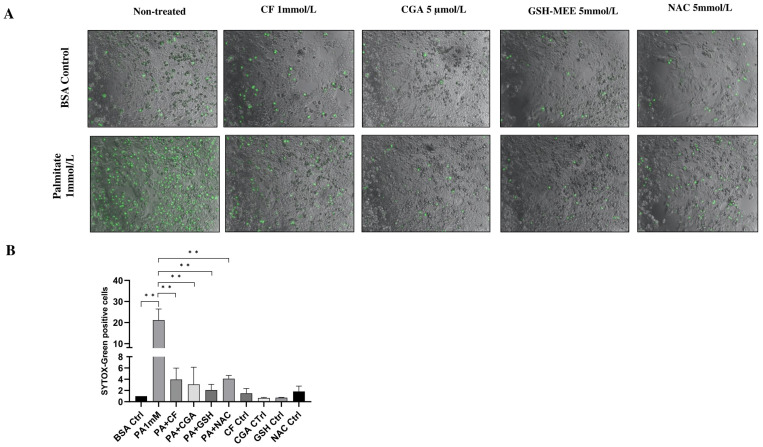
Coffee compounds prevent palmitate-induced lipotoxicity in primary rat hepatocytes. (**A**) SYTOX green staining. Green: necrotic cells. (**B**) Relative quantification of SYTOX green-positive cells compared to the BSA control. SYTOX green-positive cell counting was also normalized according to the confluency of the cell cultures. Primary rat hepatocytes were treated for 24 h with palmitate and CF: caffeine, CGA: chlorogenic acid, GSH-MEE: glutathione monoethyl ester or NAC: n-acetylcysteine. Scale bar: 100 μm. Results were obtained from three independent experiments (*n* = 3, ** *p* ≤ 0.01).

Optimal concentrations of coffee compounds were previously determined from a range of 0.05–1.5 mmol/L for CF and 2.5–200 μmol/L for CGA. The minimal protective concentration was selected for all experiments. Concentrations of GSH-MEE and NAC were selected according to previous studies in our lab [30]. These concentrations were used in all subsequent experiments. CGA has been proposed to be a strong antioxidant. Therefore, we evaluated whether this antioxidant potential was associated with protection against lipotoxicity. First, we investigated the effect of CGA on in vitro cell death triggered by palmitate at a toxic concentration (1 mmol/L). Primary rat hepatocytes were pretreated for 30 min with CF (1 mmol/L), CGA (5 μmol/L) or the antioxidants GSH-MEE (5 mmol/L) or NAC (5 mmol/L) and then co-treated with 1 mmol/L palmitate for 24 h. Supplementary Figure S1 shows the compounds used in this study. After 24 h treatment, palmitate induced significant necrosis compared to the BSA control. Palmitate-induced necrosis was significantly prevented by caffeine, CGA and the antioxidants GSH-MEE and NAC (Figure 1A,B). Specifically, the coffee compounds and NAC reduced palmitate-induced necrosis by more than 80%, whereas GSH-MEE reduced palmitate-induced necrosis by around 90% (Figure 1B). These findings demonstrate that CGA and antioxidants have protective effects against palmitate toxicity in primary rat hepatocytes.

### 3.2. Caffeine and Chlorogenic Acid Reduce Mitochondrial ROS Production

**Figure 2:**
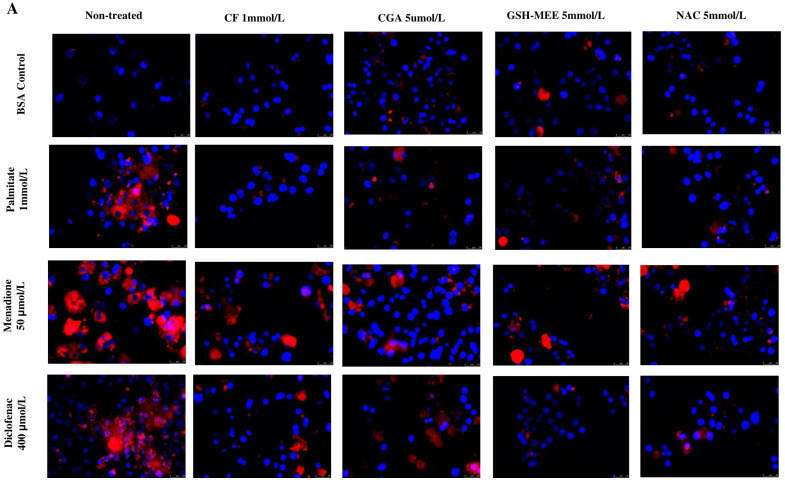

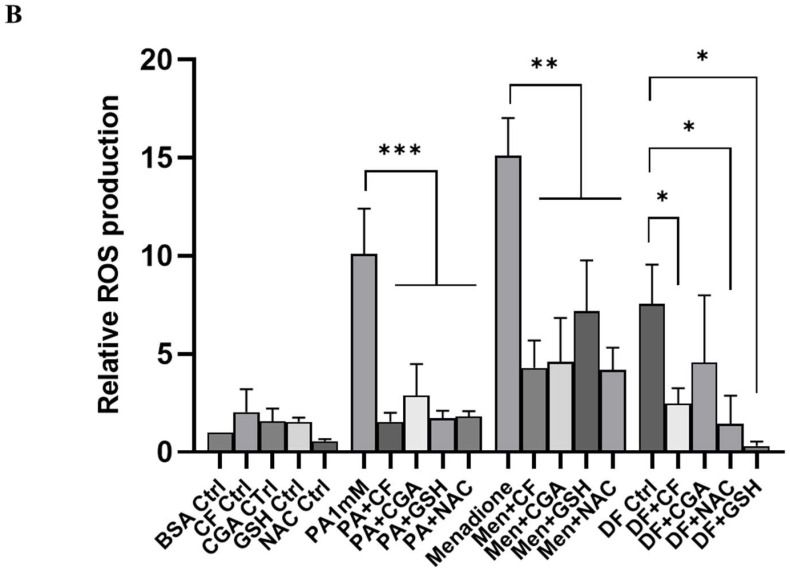
Mitochondrial ROS production is reduced by coffee compounds in palmitate-, menadione- or diclofenac-treated cells. (**A**) MitoSOX staining (red) was used to measure mitochondrial ROS production. Nuclei were visualized with DAPI (blue). (**B**) Quantification of mitochondrial ROS production. PA: palmitate 1 mmol/L, CF: caffeine 1 mmol/L, CGA: chlorogenic acid 5 μmol/L, NAC: n-acetylcysteine 5 mmol/L, GSH-MEE: glutathione monoethyl ester 5 mmol/L. Scale bar: 25 μm. Results were obtained from three independent experiments (*n* = 3). (* *p* ≤ 0.05), (** *p* ≤ 0.005), (*** *p* ≤ 0.0005).

We next investigated in more detail the relation between the antioxidant effects and the protective effects of the coffee compounds. First, mitochondrial ROS (mito-ROS) production was assessed. Menadione (50 μmol/L), a well-known superoxide generator, and diclofenac (400 μmol/L) were used as positive controls for ROS production. Hepatocytes were pretreated for 30 min with coffee compounds or antioxidant compounds and then exposed to palmitate (1 mmol/L), menadione or diclofenac for 2 h, followed by MitoSOX staining. Mito-ROS generation was significantly increased by menadione and diclofenac compared to the BSA control. Palmitate also induced ROS production in hepatocytes but to a lesser extent compared to diclofenac and menadione. CF and CGA efficiently reduced ROS production in all conditions to levels like what was observed for the antioxidants (Figure 2A). We also determined whether the reduction of ROS production by coffee compounds correlated with the reduction of caspase-3 activation by menadione, but no significant effects were observed (Figure S2). (** *p* ≤ 0.005).

These results demonstrate that both CF and CGA prevent palmitate-induced ROS production. Since oxidative stress was one of the main mechanisms triggering ER stress during lipotoxicity [5], we next explored the effects of coffee compounds on palmitate- and menadione-induced ER stress. Neither CF nor CGA significantly decreased mRNA levels of the ER stress markers CHOP or GRP78 (Figure S3, Panel A). Protein levels of GRP78 were slightly increased by menadione treatment but not with palmitate treatment. Moreover, CF and CGA did not significantly affect GRP78 levels. In contrast, GSH-MEE decreased GRP78 levels in palmitate-treated cells (PA+GSH) compared to palmitate treatment alone (PA) (Figure S3, Panel B).

### 3.3. Effect of Coffee Compounds on the Antioxidant Response in Palmitate- and Menadione-Treated Hepatocytes

**Figure 3:**
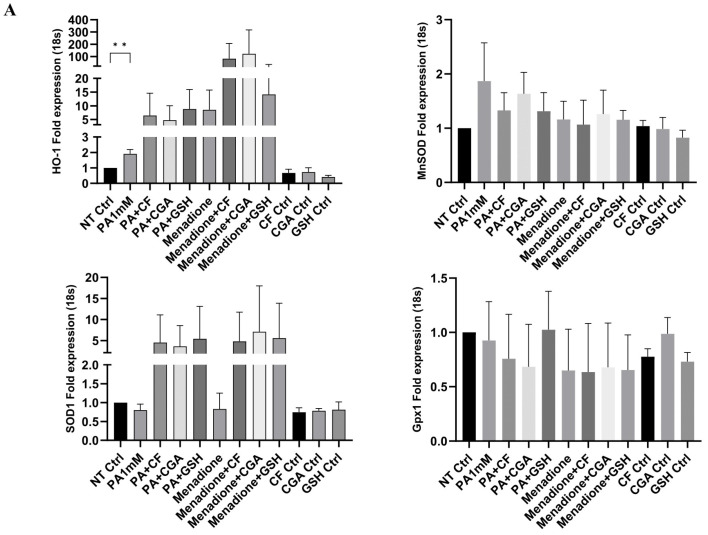

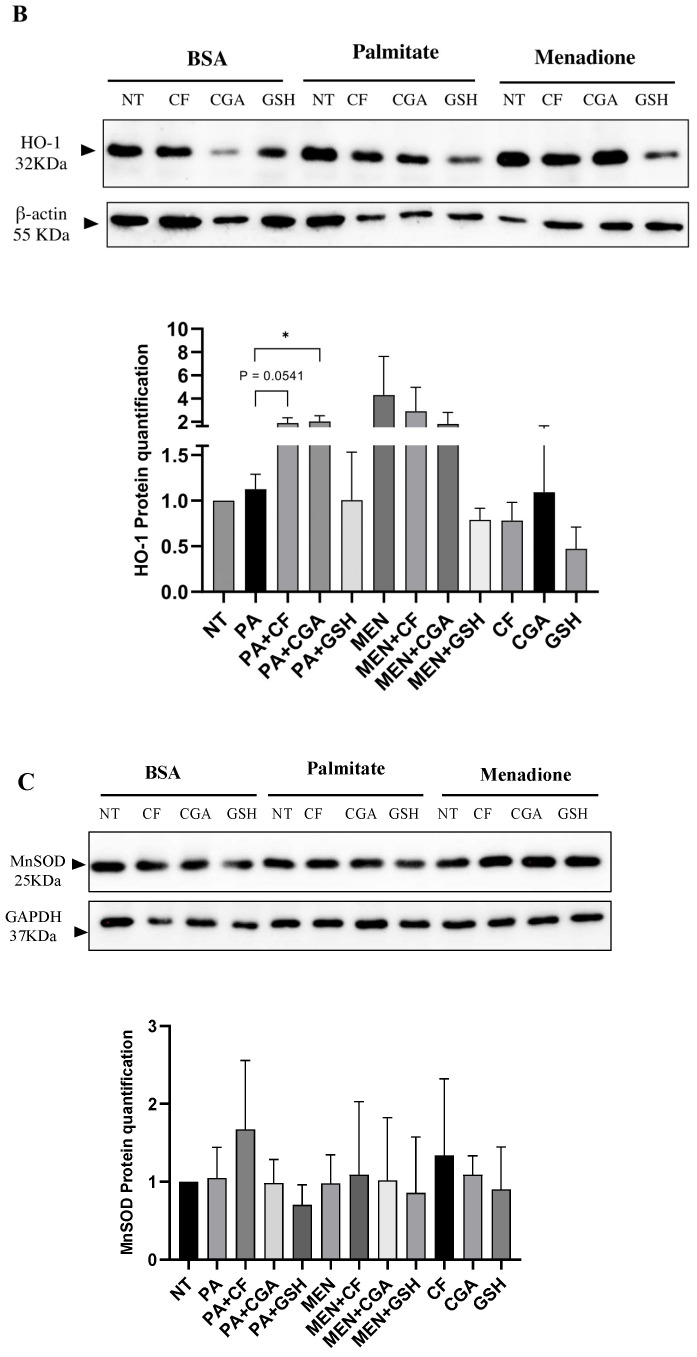

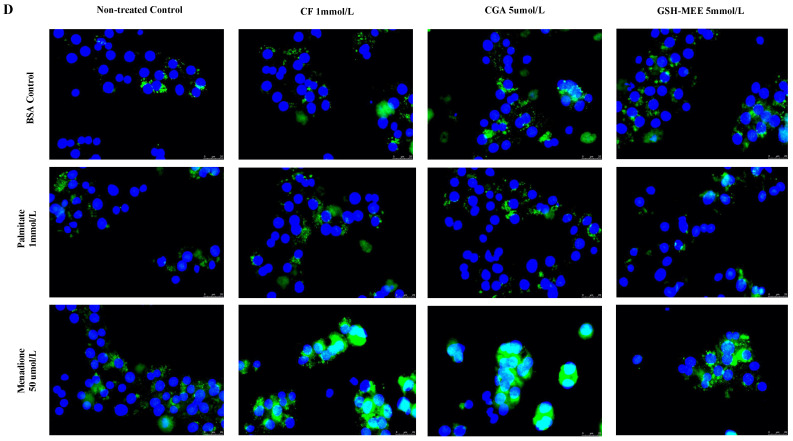
Antioxidant response is induced by coffee compounds. (**A**) mRNA levels were quantified by qRT-PCR 6 h after treatment. Results are expressed as normalized fold change relative to 18S mRNA levels for HO-1, MnSOD, SOD1 and GPx1 B. HO-1., (**B**) HO-1 and (**C**) MnSOD protein detection by Western blot and its quantification after 8 h of treatment. (**D**) Immunofluorescence for MnSOD protein. Green: MnSOD. Blue: DAPI nuclear staining. Scale bar: 25 μm. Results analysis were ob-tained from three independent experiments (*n* = 3). (* *p* ≤ 0.05, ** *p* ≤ 0.01).

Since coffee compounds reduced ROS production, we next determined whether these compounds modulated the antioxidant response by quantifying mRNA levels of different antioxidant-related genes: HO-1, SOD1 (Cu, ZnSOD), SOD2 (MnSOD) and GPx1 (Figure 3A). Palmitate treatment significantly increased HO-1 and MnSOD mRNA levels compared to the BSA-treated control but had no significant effect on SOD1 and GPx1 expression. Likewise, menadione significantly induced mRNA expression of HO-1, but no changes were observed for SOD1 and MnSOD mRNA levels. Menadione also showed a tendency to reduce GPx1 expression, but this reduction was not statistically significant. Interestingly, CF and CGA increased mRNA levels of HO-1 in both palmitate- and menadione-treated cells, but these compounds had no significant effect on SOD1, MnSOD or GPx1 mRNA expression in the absence of palmitate or menadione (Figure 3A). We next assessed protein expression levels of HO-1 and MnSOD by Western blot. Menadione significantly induced HO-1 expression compared to BSA-treated control cells, whereas palmitate did not have significant effects on HO-1 protein levels (Figure 3B). CF and CGA treatments significantly increased HO-1 protein levels in palmitate-treated cells (PA+CF, PA+CGA) compared to palmitate treatment alone. CF and CGA decreased menadione-induced HO-1 protein levels. GSH-MEE treatment reduced HO-1 protein levels both in the presence and absence of palmitate or menadione (Figure 3B). Protein expression of the Nrf2 protein, an important regulator of HO-1, was increased in the presence of CF, CGA and GSH-MEE (Figure 3C). In addition, MnSOD protein levels were slightly higher in CF-treated cells both in the absence and presence of palmitate or menadione, but these changes did not reach statistical significance. CGA and GSH-MEE had no effect on MnSOD protein levels (Figure 3D). To confirm the Western blot results, we also determined MnSOD protein expression by immunofluorescence in CF-, CGA- and GSH-MEE-treated cells (Figure 3D). CF and CGA treatments increased MnSOD expression compared to BSA-treated cells and in combination with palmitate (PA+CF and PA+CGA). MnSOD staining was also significantly higher in menadione-treated cells in the presence of CF and CGA compared to menadione-treated cells. Finally, GSH-MEE also induced MnSOD protein expression, but to a lesser extent than CF and CGA (Figure 3D).

### 3.4. Lipid Droplet Formation Is Increased by Coffee Compounds in Palmitate-Treated Cells

**Figure 4:**
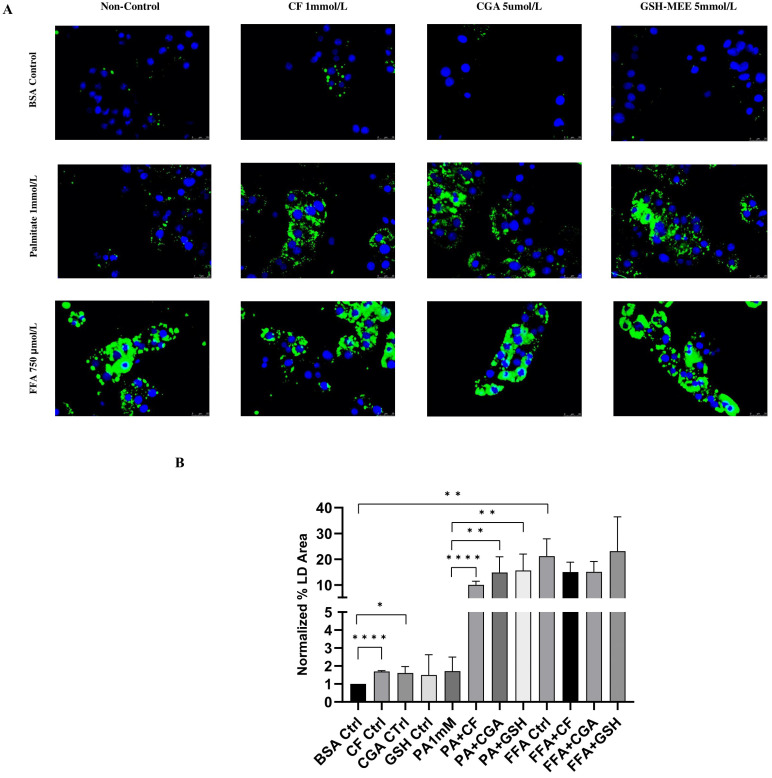
Lipid droplet formation is increased by coffee compounds in palmitate-treated cells. (**A**) BODIPY 593/50 staining was used to detect lipid droplets (green) and nuclei were visualized with DAPI (blue). (**B**) Relative % lipid droplet area (per 100 nuclei) in comparison to the BSA control. PA: palmitate 1 mmol/L, CF: caffeine 1 mmol/L, CGA: chlorogenic acid 5 μmol/L, NAC: n-acetylcysteine 5 mmol/L, GSH-MEE: glutathione monoethyl ester 5 mmol/L. Scale bar: 25 μm. Results were obtained from three independent experiments (*n* = 3). (* *p* ≤ 0.05), (** *p* ≤ 0.005), (**** *p* ≤ 0.00005).

We next investigated whether coffee compounds modulated lipid accumulation and lipid droplet formation in hepatocytes. Primary rat hepatocytes were pretreated with CF, CGA or GSH-MEE for 30 min and then treated with palmitate (1 mmol/L) or FFA mixture (750 μmol/L) for 24 h. LDs were detected by BODIPY staining. LD formation was significantly increased by FFA treatment compared to the BSA control. In contrast, palmitate treatment failed to induce significant lipid droplet formation (Figure 4A). Pre-treatment with CF, CGA or GSH-MEE significantly increased lipid droplet area (%LD area) in palmitate-treated cells compared to the BSA control and palmitate alone but did not significantly affect %LD area when combined with FFA treatment (Figure 4A,B). Remarkably, although the amounts of lipid droplets induced by the test compounds were similar to those observed in FFA-treated hepatocytes (Figure 4B), lipid droplets induced by CF, CGA and GSH-MEE were smaller in size and displayed a different distribution pattern, with a predominant localization around the cell periphery (Figure 4A).

### 3.5. Lipid Droplets Protect Against Palmitate-Induced Lipotoxicity

**Figure 5:**
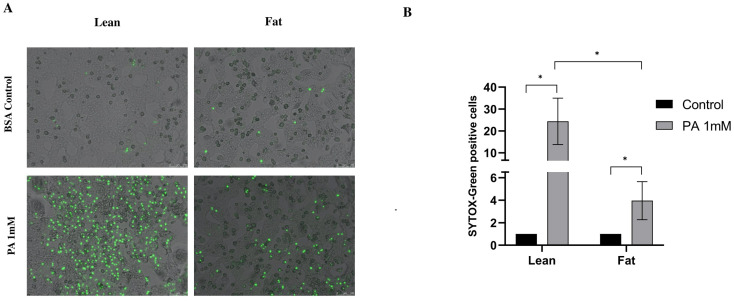

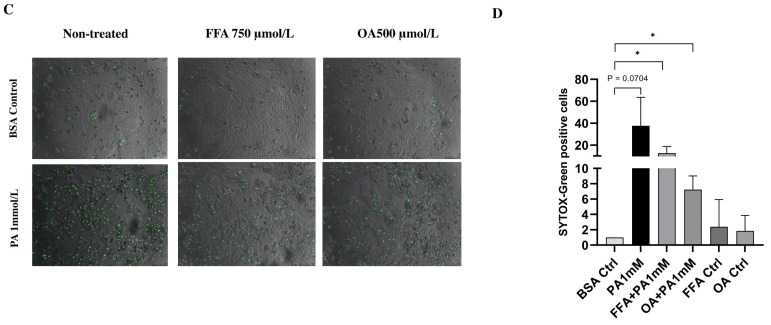
Lipid droplets protect against palmitate-induced toxicity. (**A**) SYTOX green staining for ZDF (Zucker) lean and fat hepatocytes, respectively. (**B**) Quantification of SYTOX green-positive cells. (**C**) SYTOX green staining in FFA-treated or OA-treated Wistar hepatocytes. (**D**) Quantification of SYTOX green-positive cells. PA: palmitate 1 mmol/L, OA: oleate 500 μmol/L, FFA: free fatty acid mix 750 μmol/L: (PA 250 μmol/L, OA 500 μmol/L), menadione 50 μmol/L. Scale bar: 100 μm. Results were obtained from three independent experiments (*n* = 3). (* *p* ≤ 0.05).

**Figure 6:**
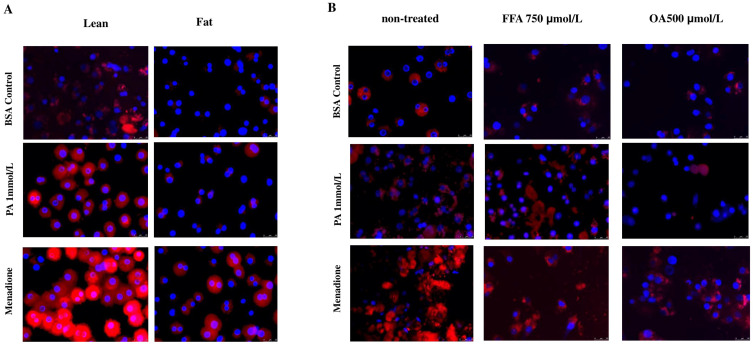

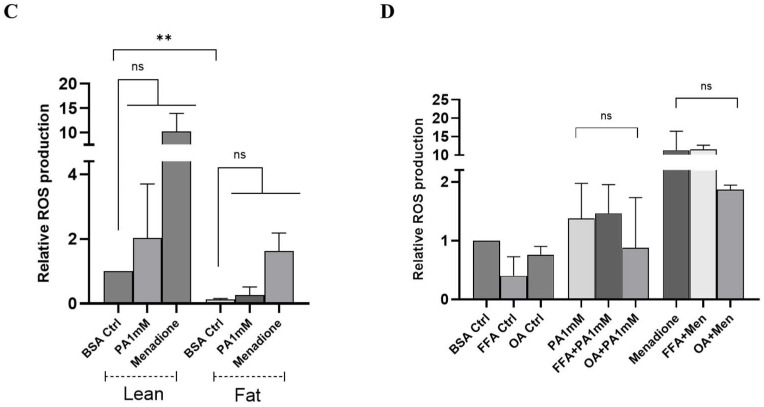
Lipid droplets are associated with reduced mitochondrial ROS production. MitoSOX staining (red) was used to measure mitochondrial ROS production. Nuclei were visualized with DAPI (blue). Mito-ROS production in (**A**) ZDF fat vs. lean hepatocytes and (**B**) FFA-pretreated or OA-pretreated Wistar hepatocytes treated with PA or menadione for 2 h Quantification of mitochondrial ROS production after Menadione treatment in (**C**) ZDF fat vs. lean hepatocytes and (**D**) FFA-pretreated or OA-pretreated Wistar hepatocytes. PA: palmitate 1 mmol/L, OA: oleate 500 μmol/L, FFA: free fatty acid mix 750 μmol/L: (PA 250 μmol/L, OA 500 μmol/L), menadione 50 μmol/L. Scale bar: 25 μm. Results were obtained from three independent experiments (*n* = 3). (** *p* ≤ 0.005), ns: no significant differences.

Lipid droplets are associated with protection against cellular stress conditions [8]. Since, in the present study, we also observed that the protective effect of CF, CGA and GSH-MEE against lipotoxicity correlated with increased lipid droplet formation, we next evaluated palmitate toxicity in primary steatotic rat hepatocytes (fat) isolated from ZDF diabetic rats (Fa/Fa), a leptin-deficient rat that develops spontaneous steatosis after 12 weeks, and hepatocytes isolated from lean Zucker rats (Fa/+). Palmitate toxicity was significantly lower in fat vs. lean hepatocytes (Figure 5A). While the number of SYTOX Green-positive cells increased around 24-fold in lean hepatocytes compared to the BSA control, this increase was only 4-fold in fat hepatocytes compared to the BSA control (Figure 5B). Next, we investigated whether the acute induction of lipid droplets also protected against palmitate toxicity. Normal Wistar rat hepatocytes were treated for 16 h with FFA mix (750 μmol/L) or oleate (OA 500 μmol/L) to induce lipid droplet formation, followed by exposure to palmitate for 24 h. As observed in ZDF fat hepatocytes, lipid droplets induced by either FFA or OA treatment protected hepatocytes from palmitate-induced cell death (Figure 5C). Necrosis was reduced by more than 60% in FFA-pretreated cells and around 80% in OA-pretreated cells compared to BSA-pretreated cells (Figure 5D). Moreover, we also determined whether caspase-3 activity triggered by menadione (after 8 h treatment) could be prevented by LDs in FFA- or OA-pretreated hepatocytes. However, neither FFA nor OA decreased caspase-3 activation in menadione-treated cells. In fact, increased caspase-3 activity was observed in FFA- and OA-pretreated hepatocytes (Figure S3, Panel A) exposed to menadione, but no necrosis was observed in FFA- or OA-pretreated hepatocytes exposed to menadione (Figure S3, Panel B). We also determined the effect of lipid droplets (ZDF and FFA- or OA-pretreated hepatocytes) on oxidative stress. Mito-ROS production triggered by palmitate or menadione was significantly lower in fat vs. lean Zucker hepatocytes (Figure 6A,C) and in both FFA- and OA-pretreated Wistar hepatocytes compared to BSA-pretreated controls (Figure 6B,D). Mito-ROS levels were significantly higher in menadione-treated hepatocytes compared to palmitate-treated hepatocytes (Figure 6C,D). These findings confirm that both long-term and short-term lipid droplet formation prevents oxidative stress induced by palmitate and menadione.

**Figure 7:**
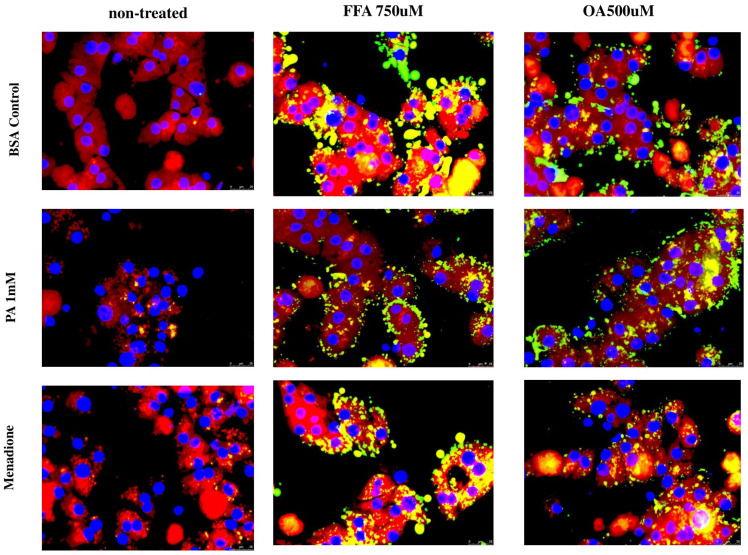
Palmitate treatment changes lipid droplet size and distribution. BODIPY 593/50 staining was used to detect lipid droplets (green) and nuclei were visualized with DAPI (blue). Lipid droplet staining in hepatocytes pretreated with FFA or OA for 16 h, followed by exposure to palmitate or menadione for 24 h. PA: palmitate 1 mmol/L, OA: oleate 500 μmol/L, FFA: free fatty acid mix 750 μmol/L: (PA 250 μmol/L, OA 500 μmol/L), menadione: Scale bar: 25 μm. Results were obtained from three independent experiments (*n* = 3).

It has been proposed that lipid droplet size and distribution determine their functional features. We observed that CF and CGA induced lipid droplet formation to an extent similar to that of the FFA mix; however, the size and distribution of these lipid droplets were different (Figure 4A), suggesting that this could be a functional feature linked to the protective mechanism of the tested coffee compounds. To investigate this in more detail, we performed lipid droplet staining in FFA- and OA-treated hepatocytes after exposure to palmitate or menadione to determine whether lipid droplet size and distribution changed during cellular stress conditions. As already observed, FFA and OA treatment induced the formation of large lipid droplets, which, in FFA-treated cells, mainly co-localized with Mitotracker, suggesting mitochondrial localization and/or association (Figure 7). No significant changes were found in %LDs area after palmitate or menadione treatment. It was noticed that FFA-induced LDs were larger than OA-induced LDs. Interestingly, palmitate treatment led to a change in the size and distribution of LDs, appearing smaller compared to FFA+BSA- or OA+BSA-treated cells, and these LDs did not co-localize with mitochondria (Figure 7). Menadione did not cause any changes in LD size and distribution, although, in OA-treated cells, increased co-staining with mitochondria was observed. 

### 3.6. Exogenous Monoacylglycerols 2-Stearoylglycerol and 2-Oleoylglycerol Have Differential Effects on Palmitate Toxicity

**Figure 8:**
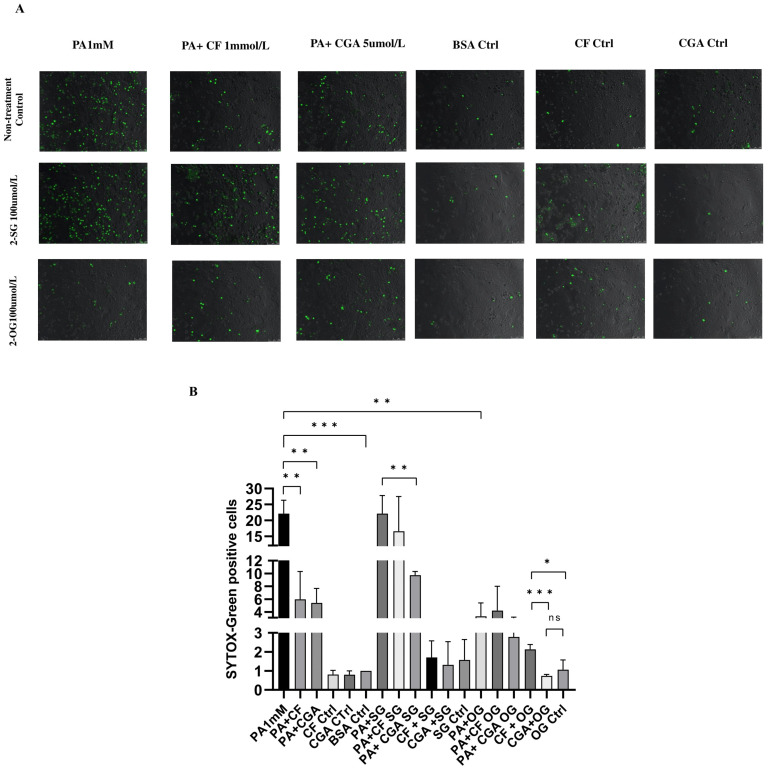
Effect of exogenous monoacylglycerol supplementation on palmitate toxicity in hepatocytes. (**A**) SYTOX green staining after 24 h of treatment with palmitate and test substances. (**B**) Quantification of SYTOX green-positive cells. PA: palmitate 1 mmol/L, CF: caffeine 1 mmol/L, CGA: chlorogenic acid 5 μmol/L, 2-SG: 2-Stearolylglycerol, 2-OG: 2-Oleoylglycerol, menadione 50 μmol/L. SYTOX green scale bar: 100 μm. Results were obtained from three independent experiments (*n* = 3). (* *p* ≤ 0.05), (** *p* ≤ 0.005), (*** *p* ≤ 0.0005), ns: no significant differences.

Based on our findings, we hypothesized that the protective effect of CF and CGA is related to a decreased intracellular saturated/unsaturated free fatty acid ratio, resulting in enhanced incorporation into lipid droplets in the form of triglycerides (TGs). Monoacylglycerols are important intermediates in TG synthesis. Therefore, in the next set of experiments, we modulated TG synthesis using the exogenous supplementation of two types of monoacylglycerol: monounsaturated 2-oleoylglycerol (2-OG, 100 μmol/L) and saturated 2-stearoylglycerol (2-SG, 100 μmol/L), according to previously optimized conditions in our lab [31]. The treatment of hepatocytes with palmitate with or without 2-SG (PA+2-SG) caused significant necrosis compared to the BSA control. In contrast, the treatment of hepatocytes with 2-OG effectively prevented palmitate-induced cell death (Figure 8A). Moreover, the protective effects of CF and CGA were abolished by 2-SG (Figure 8A), as shown by a significant increase in the number of SYTOX Green-positive cells when 2-SG was added (PA+CF+2-SG and PA+CGA+2-SG) (Figure 8B). In contrast, the combination of 2-OG with both CF and CGA enhanced protection against palmitate toxicity. Moreover, the effects of 2-SG and 2-OG correlated with ROS production. In particular, 2-SG treatment resulted in higher ROS production in combination with palmitate, palmitate plus CF (PA+CF) and palmitate plus CGA (PA+CGA), whereas 2-OG decreased ROS production in both menadione- and palmitate-treated hepatocytes (Figure S5).

**Figure 9:**
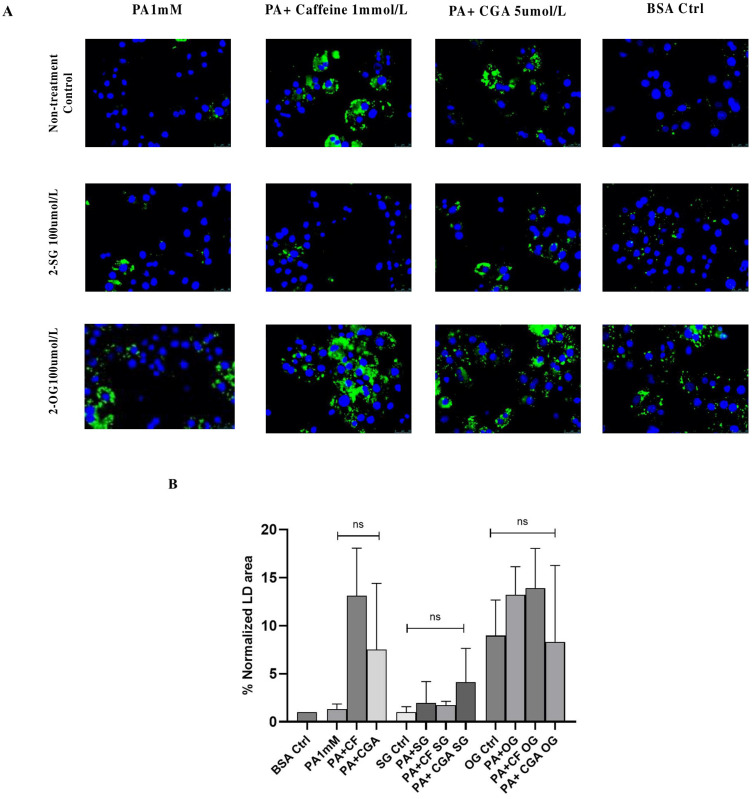
Effect of exogenous monoacylglycerols on lipid droplet formation in palmitate-treated hepatocytes. BODIPY 593/50 staining was used to detect lipid droplets (green) and nuclei were visualized with DAPI (blue). (**A**) Lipid droplet staining in 2-SG- or 2-OG-treated hepatocytes in combination with PA, PA+C or PA+CGA for 24 h. (**B**) Relative %lipid droplet area (per 100 nuclei) with respect to the BSA control. PA: palmitate 1 mmol/L, CF: caffeine 1 mmol/L, CGA: chlorogenic acid 5 μmol/L, 2-SG: 2-Stearolylglycerol, 2-OG: 2-Oleoylglycerol, menadione 50 μmol/L. Scale bar: 25 μm. Results were obtained from three independent experiments (*n* = 3). ns: no significant differences.

These findings support the notion that CF and CGA protection depends on the ability to stimulate LD formation, which can be affected by the intracellular ratio of saturated to unsaturated fatty acids. We next investigated exogenous 2-SG and 2-OG monoacylglycerol modulate intracellular LD formation in palmitate-treated hepatocytes in the absence and presence of coffee compounds. LD formation induced by CF (PA+CF) and CGA (PA+CGA) was significantly decreased by 2-SG treatment, and a very low number of LDs were detected in PA+2-SG-treated cells. The monounsaturated monoacylglycerol 2-OG increased LD formation in both palmitate- and BSA-treated cells. Likewise, 2-OG enhanced LD formation induced by CF and CGA (Figure 9A). These results demonstrate that monounsaturated monoacylglycerols effectively stimulate LD formation in the presence of palmitate, thus attenuating lipotoxicity. In addition, our results also show that saturated monoacylglycerol 2-SG interferes with the protective effects of CF and CGA.

### 3.7. SCD-1 Inhibition Is Associated with Increased Palmitate Toxicity

**Figure 10:**
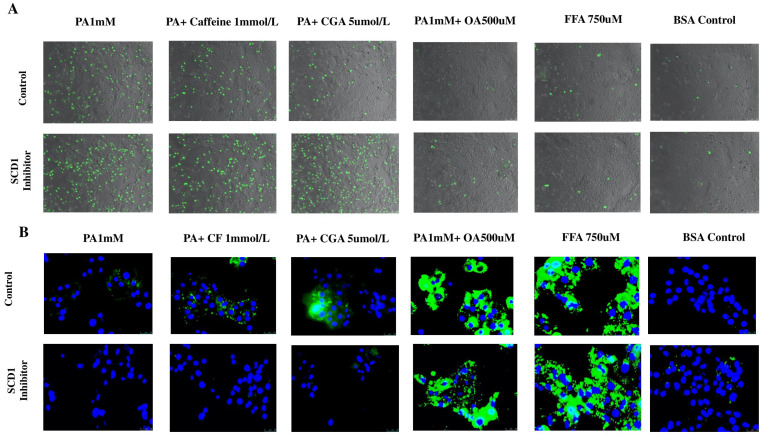

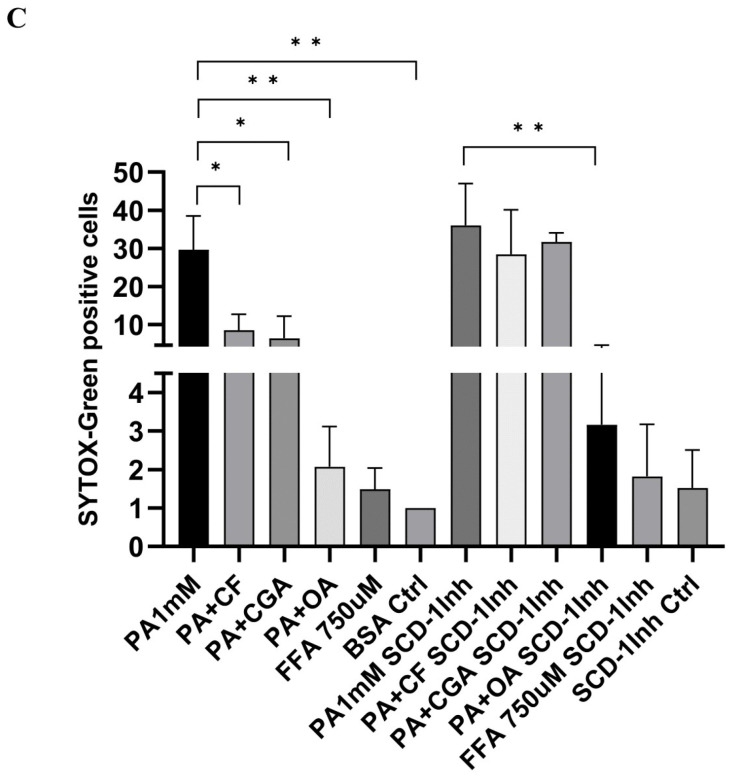
The protective effect of CF and CGA against lipotoxicity is dependent on SCD-1 activity. (**A**) SYTOX green staining in palmitate-treated hepatocytes in combination with SCD-1 inhibitor. (**B**) BODIPY 593/50 staining was used to detect lipid droplets (green) and nuclei were visualized with DAPI (blue). (**C**) Quantification of SYTOX green-positive cells. PA: palmitate 1 mmol/L, CF: caffeine 1 mmol/L, CGA: chlorogenic acid 5 μmol/L, OA: oleate 500 μmol/L, FFA: free fatty acid mix 750 μmol/L: (PA 250 μmol/L, OA 500 μmol/L), SCD-1 inhibitor: Stearoyl-CoA Desaturase 1 inhibitor 10 μmol/L. SYTOX green scale bar: 100 μm, BODIPY scale bar: 25 μm. Results were obtained from three independent experiments (*n* = 3). (* *p* ≤ 0.05), (** *p* ≤ 0.005).

Since our results support the hypothesis that the protective effect of CF and CGA against lipotoxicity is due to the increased incorporation of palmitate into LDs, we next evaluated the effect of a selective chemical inhibitor of SCD-1, the enzyme involved in the synthesis of monounsaturated fatty acyl-CoA. Specifically, SCD-1 catalyzes the conversion of palmitate (C16:0) into palmitoleic (16:1∆9), facilitating efficient TG synthesis and subsequent incorporation into LDs. After 24 h of treatment with palmitate or palmitate plus SCD-1 inhibitor, similar levels of necrosis were observed, indicating that SCD-1 inhibition did not significantly enhance palmitate toxicity. In contrast, SCD-1 inhibition abolished almost completely the protective effect of CF and CGA. SCD-1 inhibition had no effect on OA- and FFA-treated cells. Moreover, SCD-1 inhibition did not cause significant necrosis in PA+OA- or FFA-treated hepatocytes (Figure 10A,C). Likewise, SCD-1 inhibition blocked LD formation induced by CF and CGA in palmitate-treated cells but had no effect on the increased LD formation induced by OA and FFA treatment, indicating that protection against palmitate toxicity correlates with LD formation (Figure 10B). These observations confirm that SCD-1 activity mediates the protective effects of CF and CGA against palmitate lipotoxicity and suggest that SCD-1 is a likely molecular target of these two coffee compounds in hepatocytes.

## 4. Discussion

Coffee constitutes an important source of dietary antioxidants since it contains several bioactive compounds with biological properties, including antioxidant and anti-inflammatory effects [17]. In fact, coffee consumption is associated with an improvement in redox status [15,16] and a lower risk of MASLD development and progression [12,32]. The beneficial effects of coffee on MASLD have been associated with CF, the most abundant compound present in the beverage [33]. However, decaffeinated coffee also improved clinical outcomes in chronic liver disease and steatosis [34]. Decaffeinated coffee protected against MASH in an experimental mouse model by preventing inflammation and maintaining intestinal barrier function [35], effects that could be explained by the antioxidant capacity of bioactive compounds such as CGA or coffee polyphenols. CGA (or 5-O-caffeoylquinic acid, CQA) is one of the major polyphenols in the human diet. It is a strong antioxidant and reduces the risk of metabolic and chronic diseases [36].

Hepatic lipotoxicity refers to the detrimental effects of excessive exposure to (toxic) lipid species, in particular (saturated) FFAs, such as palmitate. Oxidative stress is a key mechanism of lipotoxicity in hepatocytes and ultimately leads to cell death and inflammation [5]. Reducing oxidative stress using antioxidants has been explored as a potential therapy for MASLD [37]. In this study, we explored the mechanisms of the protective effects of CF and CGA against palmitate-induced lipid toxicity in primary rat hepatocytes, with particular emphasis on their antioxidant potential and their modulation of lipid droplet formation during cellular stress conditions. We observed that CF and CGA reduced mitochondrial ROS production, with the significant induction of HO-1 expression (mRNA and protein), especially in palmitate-treated hepatocytes. HO-1 is an antioxidant enzyme and its expression is induced by oxidative stress and other cellular stressors to counteract oxidative stress. A number of natural compounds have been reported to induce HO-1 [38,39]. Increased HO-1 expression has been observed in mice on a high-fat diet and palmitate-treated HepG2 cells. Moreover, in the same study, the knockdown of HO-1 resulted in increased oxidative stress induced by palmitate, demonstrating that HO-1 plays an important role in the antioxidant response to reduce lipid toxicity derived from saturated fatty acids (SFAs) in the liver [40]. Consequently, HO-1 induction by CF and CGA contributes to protection against lipotoxicity. We also observed Nrf2 induction after CF or CGA treatment in combination with palmitate. HO-1 expression is regulated by the Nrf2-antioxidant response [38]. Coffee compounds have been reported to modulate Nrf2 activation, resulting in an improvement of the hepatic antioxidant system [41,42]. Based on this, we conclude from our experimental findings that CF and CGA promote the antioxidant response via Nrf2 activation and subsequent HO-1 induction, conferring protection against palmitate toxicity. CF and CGA have structural differences, but both compounds protect against lipotoxicity and ROS production. However, the protective concentration of CGA (5 μmol/L) was 200 times lower compared to CF (1 mmol/L), indicating the greater antioxidant potential of CGA, most likely due to its polyphenolic structure. CGAs represent a group of hydroxyl cinnamic esters with ROS scavenging capacity, also known as chain-breaking antioxidants [43].

Regarding the antioxidant properties of coffee compounds, both CF and CGA have shown a high affinity to scavenge hydroxyl, peroxyl and superoxide radicals, which might be explained by their molecular capacity to react either by the hydrogen atom transfer (HAT) or single-electron transfer mechanism (SET). In fact, due to the chemical structure of CF and CGA and based on some in vitro antioxidant assay evidence, a combination of both antioxidant reaction mechanisms is plausible [44].

We also used the glutathione (GSH) precursor GSH-MEE, which effectively decreased mito-ROS production and protected hepatocytes from palmitate toxicity but did not induce the Nrf2 antioxidant response. This result implies that counteracting oxidative stress, even via different mechanisms, is enough to prevent palmitate toxicity: GSH-MEE protects by increasing intracellular GSH levels, whereas CF and CGA act as Nrf2 inducers.

Interestingly, we observed that the protective effect of the coffee compounds CF and CGA as well as GSH-MEE correlated with significantly increased LD formation. This correlation suggests that LDs play a role in protection against lipotoxicity. This observation has not been reported before. In fact, a number of dietary antioxidants have been shown to improve MASLD while reducing lipid accumulation due to the reduction of lipid synthesis, ROS production and ER stress [45]. For instance, CF was shown to reduce lipid accumulation via the downregulation of lipogenesis-related genes and ER stress in zebrafish larvae [46]. Likewise, CGA treatment alleviated NASH in mice and decreased hepatic lipid accumulation [47]. In contrast to these studies, we observed a protective effect of increased LD accumulation against palmitate toxicity. We also evaluated the effect of CF and CGA treatment on lipid accumulation induced by FFAs (PA+OA) but did not observe any significant differences in total LDs (Figure 6). In this regard, differential effects on lipid accumulation have been reported for saturated versus unsaturated FFAs: the unsaturated FFA oleic acid causes more lipid accumulation (LDs and TGs) than the saturated FFA palmitate. Moreover, we observed that lipid accumulation is inversely correlated with apoptotic cell death: palmitate induced significant apoptosis in HepG2 cells but not lipid accumulation, whereas the opposite was observed for oleate. A possible explanation is that PA and OA treatments result in different FFA profiles, particularly different unsaturated/saturated fatty acid ratios [48]. In support of this, it has been reported that PA and OA have different effects on LD distribution and interaction with other organelles such as mitochondria, explaining the different effects of PA and OA on mitochondrial function [49]. These studies correlate with our findings, in which OA and FFA showed no toxic effects on primary rat hepatocytes but rather protected against palmitate-induced toxicity.

We therefore propose that the observed increase in LDs by CF and CGA treatment is due to a switch in the unsaturated/saturated FFA ratio, resulting in the enhanced incorporation of palmitate into LDs and less toxicity. This is supported by the effect of 2-OG (unsaturated) monoacylglycerol, demonstrating that the stimulation of TG synthesis by 2-OG protected against lipotoxicity and increased LDs in palmitate-treated hepatocytes. 2-OG also improved the protective effect of CF and CGA. In contrast, treatment with 2-SG (saturated) monoacylglycerol abolished LD formation in the presence of CF and CGA and inhibited their protective effect, supporting the hypothesis that the protective effect of CF and CGA is due to a decreased saturated/unsaturated FFA ratio. Lipotoxicity mainly results from the toxic effects of SFAs [50] by increasing the formation of intermediate toxic lipid species (e.g., ceramides), leading to increased ROS production, lipid peroxidation, ER stress and mitochondrial dysfunction [51]. Unlike unsaturated FFAs, long-chain and very-long-chain (C16–C22) SFAs cause lipotoxicity and lower lipid accumulation, most likely because they are less prone to being incorporated into LDs in the form of TGs [52]. Therefore, it is plausible that mechanisms leading to the increased incorporation of SFAs into LDs result in less lipid toxicity.

LDs are considered important organelles that are important in maintaining cellular homeostasis and play a role in the cellular response to several metabolic stress factors [8,53], e.g., nutrient starvation and ER stress [54,55,56]. A potential antioxidant role of LDs was described for Drosophila larvae fed a diet rich in polyunsaturated fatty acids (PUFAs), demonstrating that LDs prevent lipid peroxidation by sequestering FFAs [57]. We also observed that treatment with FFA and coffee compounds or the antioxidant NAC minimally increased caspase-3 activation (Figure 2 and Figure S2), which might be related to their antioxidant effect, but no significant necrosis was detected after 24 h in these conditions. This phenomenon might be explained by an adaptation mechanism as it has been shown that cell survival might be regulated via ROS production and that decreasing ROS production by antioxidants like NAC results in higher caspase-3 activity [58].

The role of LDs in lipotoxicity has not been explored very well in hepatocytes; however, it has been shown that the composition and intracellular distribution of LDs reflect metabolic alterations [59]. Lipid accumulation is heterogeneous within a cell population, with some cells storing more lipids than others, suggesting the compartmentalization of lipid storage, and, consequently, cells containing high levels of lipids may protect other cells from lipotoxic effects [60]. In our experiments, we also noticed that LD distribution in primary rat hepatocytes was heterogeneous both in FFA- and OA-treated cells as well as in CF- and CGA-treated cells (Figure 7, Figure 8, Figure 9 and Figure 10).

Our experiments showed that CF and CGA increased LD accumulation in palmitate-treated hepatocytes, most likely via a mechanism dependent on SCD-1 activity (Figure 10). We also demonstrated that the protective effect of coffee compounds correlates with a decreased ratio of saturated/unsaturated FFAs, which is dependent on SCD-1 activity. Moreover, experiments in Sprague-Dawley rats fed a cocoa-based high-fat diet (HFD) plus fructose demonstrated that both CF supplementation and the supplementation of green coffee extracts (GCEs), which are high in polyphenols and CGA, did not prevent hepatic steatosis but did have an effect on the hepatic TG fatty acid composition: HFD increased the total amount of FFAs (palmitic acid, stearic acid, oleic acid and MUFAs), whereas CF supplementation increased the monounsaturated fatty acids (MUFAs) content compared to an HFD. Interestingly, in this study, CF and GCE decreased the content of diacylglycerol (DAG) and ceramides, whereas the expression of SCD-1, a rate-limiting enzyme involved in the synthesis of MUFAs, was increased. No significant effect on other lipogenesis-related genes (SREBP1c, FAS) was observed [61]. In experimental models of steatosis in *C. elegans* and HFD-fed mice, caffeine promoted conversion from palmitic acid to palmitoleic acid by inducing the expression of SCD-1, resulting in changes in fatty acid composition and increased monounsaturated/saturated FFA ratios [62]. These studies are in line with our experiments and support the notion that coffee compounds decrease lipotoxicity by decreasing the saturated/unsaturated FFA ratio. Likewise, SCD-1 might be a suitable therapeutic target to counteract hepatic lipotoxicity since this enzyme is necessary to maintain the MUFA pool, which consists of the major lipid species incorporated into LDs in the form of TGs [63].

In this regard, the protective role of SCD-1 has been reported before in human mesenchymal stromal cells where increasing SCD-1 expression decreased the SFA/MUFA ratio and reduced palmitate toxicity and inflammation [64]. Likewise, the downregulation of SCD-1 increased palmitate-induced lipotoxicity in insulin-producing ß-cells [65] and human trophoblast cells [66]. Moreover, SCD-1 deficiency in mice causes an increase in fatty acid oxidation and increased SFA/MUFA ratios [67], which can augment palmitate-induced mitochondrial dysfunction. SCD-1 is positively regulated by lipogenesis-related transcription factors such as liver X receptor (LXR), sterol regulatory element binding protein-1c (SREBP-1c), carbohydrate response element binding protein (CHREBP) [63] and peroxisome proliferator-activated receptors (PPARs) [68]. However, the regulation of SCD-1 by coffee compounds still needs to be elucidated as well as its regulation by oxidative stress and the antioxidant response.

Lipid droplet size and distribution have been associated with functional effects that can influence lipid metabolism during cellular stress conditions. Recent findings demonstrated that LD size determines the balance between lipolysis and lipophagy, two pathways involved in the catabolism of LDs and lipids. LD diameters range from 60 nm to well over 5 μm in steatotic conditions. Cytoplasmic lipases (e.g., ATGL) preferentially degrade large LDs in hepatocytes, whereas small LDs are targeted for lipophagy catabolism [69]. Autophagy, particularly lipophagy, has been reported to be involved in the response against palmitate toxicity and is an important pathway for LD catabolism [70,71,72,73]. Caffeine also stimulates hepatic lipid metabolism via the activation of hepatic autophagy and the downregulation of mTOR with the concomitant activation of mitochondrial β-oxidation [74,75]. Similarly, caffeic acid, another type of coffee polyphenol, has been reported to increase autophagy in the livers of mice fed an HFD [76]. We hypothesize that CF and CGA stimulate lipophagy, but more detailed studies are necessary to elucidate these mechanisms and their potential as a therapy for MASLD. Moreover, the mechanisms that link LD metabolism and lipophagy are still unclear.

The regulation of LD catabolism by changing LD size could also be a mechanism to counteract lipotoxicity [77]. Since coffee compounds also modulate LD size, this could be another mechanism by which coffee compounds protect against lipotoxicity. Interestingly, it has been also shown that SCD-1 regulates LD size and phospholipid composition, and SCD-1 activity is essential to increase the size of LDs.

## 5. Conclusions

The present study showed that the coffee compounds CF and CGA protect against lipotoxicity in hepatocytes via a mechanism dependent on SCD-1 activity, indicating that SCD-1 can be a potential molecular target. Moreover, we hypothesized that the protective effects of CF and CGA are related to reducing oxidative stress and increased lipid droplet formation. Furthermore, we proposed that LDs might reduce oxidative stress since oxidative stress-related toxicity in hepatocytes can be prevented by increasing lipid droplet content. Understanding the metabolism of lipid droplet formation and distribution is key to understanding lipotoxicity and may lead to the identification of novel targets for the treatment of MASLD.

## Data Availability

The data supporting the reported results are stored on the department’s secured Y-drive and available upon request to the corresponding author.

## Supplementary Materials

The following supporting information can be downloaded at https://www.mdpi.com/article/10.3390/antiox14020175/s1: Table S1. List of primers and probes; Figure S1. Coffee compounds and antioxidants used in this study. A. Caffeine, CF (1,3,7-Trimethylxanthine). B. Chlorogenic acid, CGA (1,4,5-Trihydroxycyclohexanecarboxylic acid). C. Glutathione monoethyl ester, GSH-MEE. D. N-acetylcysteine, NAC. E. Saturated 2-SG (2-Stearolylglycerol). F. Monounsaturated fatty acid 2-OG (2-Oleoylglycerol).; Figure S2. Caspase-3 activity after 16 h of treatment with menadione and CF: caffeine, CGA: chlorogenic acid or NAC.; Figure S3. Effect of coffee compounds on ER stress markers. A. mRNA levels were quantified by qRT-PCR 6 h after treatment with compounds. GRP78 and CHOP expression levels are expressed as normalized fold change relative to 18S mRNA levels. B. GRP78 and C. Nrf2 protein detection by Western blot and quantification after 8 h of treatment with compounds.; Figure S4. A. Caspase-3 activity after 16 h of treatment with menadione and FFA mix or OA. B. SYTOX green staining after 24 h of treatment with menadione and FFA mix or OA.; Figure S5. Effect of exogenous monoacylglycerol supplementation on palmitate toxicity and ROS production in hepatocytes. MitoSOX staining (red) was used to measure Mito-ROS production. Nuclei were visualized with DAPI (blue). Mito-ROS was measured after 2 h of treatment with palmitate and test substances. PA: palmitate 1 mmol/L, CF: caffeine 1 mmol/L, CGA: chlorogenic acid 5 μmol/L, 2-SG: 2-Stearolylglycerol, 2-OG: 2-Oleoylglycerol, menadione 50 μmol/L. SYTOX green scale bar: 100 μm. MitoSOX scale bar: 25 μm. Results were obtained from three independent experiments (*n* = 3).; Figure S6. SYTOX green staining in SCD-1-treated hepatocytes in combination with CF: caffeine or CGA: chlorogenic acid.

## References

[1] Kim D., Konyn P., Sandhu K.K., Dennis B.B., Cheung A.C., Ahmed A. (2021). Metabolic Dysfunction-Associated Fatty Liver Disease Is Associated with Increased All-Cause Mortality in the United States. J. Hepatol..

[2] Rinella M.E., Lazarus J.V., Ratziu V., Francque S.M., Sanyal A.J., Kanwal F., Romero D., Abdelmalek M.F., Anstee Q.M., Arab J.P. (2023). A Multi-Society Delphi Consensus Statement on New Fatty Liver Disease Nomenclature. Hepatology.

[3] Eslam M., Newsome P.N., Sarin S.K., Anstee Q.M., Targher G., Romero-Gomez M., Zelber-Sagi S., Wai-Sun Wong V., Dufour J.F., Schattenberg J.M. (2020). A New Definition for Metabolic Dysfunction-Associated Fatty Liver Disease: An International Expert Consensus Statement. J. Hepatol..

[4] Xu X., Poulsen K.L., Wu L., Liu S., Miyata T., Song Q., Wei Q., Zhao C., Lin C., Yang J. (2022). Targeted Therapeutics and Novel Signaling Pathways in Non-Alcohol-Associated Fatty Liver/Steatohepatitis (NAFL/NASH). Signal Transduct. Target. Ther..

[5] Arroyave-Ospina J.C., Wu Z., Geng Y., Moshage H. (2021). Role of Oxidative Stress in the Pathogenesis of Non-Alcoholic Fatty Liver Disease: Implications for Prevention and Therapy. Antioxidants.

[6] Geng Y., Faber K.N., de Meijer V.E., Blokzijl H., Moshage H. (2021). How Does Hepatic Lipid Accumulation Lead to Lipotoxicity in Non-Alcoholic Fatty Liver Disease?. Hepatol. Int..

[7] Listenberger L.L., Han X., Lewis S.E., Cases S., Farese R.V., Ory D.S., Schaffer J.E. (2003). Triglyceride Accumulation Protects against Fatty Acid-Induced Lipotoxicity. Proc. Natl. Acad. Sci. USA.

[8] Jarc E., Petan T. (2019). Lipid Droplets and the Management of Cellular Stress. Yale J. Biol. Med..

[9] Tong X., Stein R. (2021). Lipid Droplets Protect Human B-Cells From Lipotoxicity-Induced Stress and Cell Identity Changes. Diabetes.

[10] Plötz T., Hartmann M., Lenzen S., Elsner M. (2016). The Role of Lipid Droplet Formation in the Protection of Unsaturated Fatty Acids against Palmitic Acid Induced Lipotoxicity to Rat Insulin-Producing Cells. Nutr. Metab..

[11] Che T., Yan C., Tian D., Zhang X., Liu X., Wu Z. (2021). The Association Between Sleep and Metabolic Syndrome: A Systematic Review and Meta-Analysis. Front. Endocrinol..

[12] Sewter R., Heaney S., Patterson A. (2021). Coffee Consumption and the Progression of Nafld: A Systematic Review. Nutrients.

[13] Kaur M., Murugesan S., Singh S., Uy K.N., Kaur J., Mann N., Sekhon R.K. (2023). The Influence of Coffee on Reducing Metabolic Dysfunction-Associated Steatotic Liver Disease in Patients With Type 2 Diabetes: A Review. Cureus.

[14] Salvoza N., Giraudi P.J., Tiribelli C., Rosso N. (2022). Natural Compounds for Counteracting Nonalcoholic Fatty Liver Disease (NAFLD): Advantages and Limitations of the Suggested Candidates. Int. J. Mol. Sci..

[15] Agudelo-Ochoa G.M., Pulgarín-Zapata I.C., Velásquez-Rodriguez C.M., Duque-Ramírez M., Naranjo-Cano M., Quintero-Ortiz M.M., Lara-Guzmán O.J., Muñoz-Durango K. (2016). Coffee Consumption Increases the Antioxidant Capacity of Plasma and Has No Effect on the Lipid Profile or Vascular Function in Healthy Adults in a Randomized Controlled Trial. J. Nutr..

[16] Martini D., Del Bo’ C., Tassotti M., Riso P., Rio D.D.e.l., Brighenti F., Porrini M. (2016). Coffee Consumption and Oxidative Stress: A Review of Human Intervention Studies. Molecules.

[17] Nuhu A.A. (2014). Bioactive Micronutrients in Coffee: Recent Analytical Approaches for Characterization and Quantification. ISRN Nutr..

[18] Metro D., Cernaro V., Santoro D., Papa M., Buemi M., Benvenga S., Manasseri L. (2017). Beneficial Effects of Oral Pure Caffeine on Oxidative Stress. J. Clin. Transl. Endocrinol..

[19] Barcelos R.P., Lima F.D., Carvalho N.R., Bresciani G., Royes L.F. (2020). Caffeine Effects on Systemic Metabolism, Oxidative-Inflammatory Pathways, and Exercise Performance. Nutr. Res..

[20] Liang N., Kitts D.D. (2015). Role of Chlorogenic Acids in Controlling Oxidative and Inflammatory Stress Conditions. Nutrients.

[21] Jiang H., He K., Luo X., Zhang M., Shao J., Gan L., Lin Y., Qin C., Zhang H., Wei Q. (2022). Chlorogenic Acid Attenuates Inflammation, Oxidative Stress, Apoptosis and Protects Head Kidney Macrophage of Yellow Catfish from Ammonia Toxicity. Aquac. Res..

[22] Chen Z., Yang Y., Mi S., Fan Q., Sun X., Deng B., Wu G., Li Y., Zhou Q., Ruan Z. (2019). Hepatoprotective Effect of Chlorogenic Acid against Chronic Liver Injury in Inflammatory Rats. J. Funct. Foods.

[23] Farias-Pereira R., Park C.S., Park Y. (2019). Mechanisms of Action of Coffee Bioactive Components on Lipid Metabolism. Food Sci. Biotechnol..

[24] Arroyave-Ospina J.C., Buist-Homan M., Schmidt M., Moshage H. (2023). Protective Effects of Caffeine against Palmitate-Induced Lipid Toxicity in Primary Rat Hepatocytes Is Associated with Modulation of Adenosine Receptor A1 Signaling. Biomed. Pharmacother..

[25] Yang L., Wei J., Sheng F., Li P. (2019). Attenuation of Palmitic Acid–Induced Lipotoxicity by Chlorogenic Acid through Activation of SIRT1 in Hepatocytes. Mol. Nutr. Food Res..

[26] Zhang Y., Miao L., Zhang H., Wu G., Zhang Z., Lv J. (2018). Chlorogenic Acid against Palmitic Acid in Endoplasmic Reticulum Stress-Mediated Apoptosis Resulting in Protective Effect of Primary Rat Hepatocytes. Lipids Health Dis..

[27] Geng Y., Hernández Villanueva A., Oun A., Buist-Homan M., Blokzijl H., Faber K.N., Dolga A., Moshage H. (2020). Protective Effect of Metformin against Palmitate-Induced Hepatic Cell Death. Biochim. Biophys. Acta—Mol. Basis Dis..

[28] Moshage H., Casini A., Lieber C.S. (1990). Acetaldehyde Selectively Stimulates Collagen Production in Cultured Rat Liver Fat-storing Cells but Not in Hepatocytes. Hepatology.

[29] Schoemaker M.H., Ros J.E., Homan M., Trautwein C., Liston P., Poelstra K., van Goor H., Jansen P.L.M., Moshage H. (2002). Cytokine Regulation of Pro- and Anti-Apoptotic Genes in Rat Hepatocytes: NF-KappaB-Regulated Inhibitor of Apoptosis Protein 2 (CIAP2) Prevents Apoptosis. J. Hepatol..

[30] de la Rosa L.C., Vrenken T.E., Buist-Homan M., Faber K.N., Moshage H. (2015). Metformin Protects Primary Rat Hepatocytes against Oxidative Stress-Induced Apoptosis. Pharmacol. Res. Perspect..

[31] Geng Y., Arroyave-Ospina J.C., Buist-Homan M., Plantinga J., Olinga P., Reijngoud D.J., Van Vilsteren F.G.I., Blokzijl H., Kamps J.A.A.M., Moshage H. (2023). Differential Effects of Oleate on Vascular Endothelial and Liver Sinusoidal Endothelial Cells Reveal Its Toxic Features in Vitro. J. Nutr. Biochem..

[32] Kositamongkol C., Kanchanasurakit S., Auttamalang C., Inchai N., Kabkaew T., Kitpark S., Chaiyakunapruk N., Duangjai A., Saokaew S., Phisalprapa P. (2021). Coffee Consumption and Non-Alcoholic Fatty Liver Disease: An Umbrella Review and a Systematic Review and Meta-Analysis. Front. Pharmacol..

[33] Vargas-Pozada E.E., Ramos-Tovar E., Acero-Hernández C., Cardoso-Lezama I., Galindo-Gómez S., Tsutsumi V., Muriel P. (2022). Caffeine Mitigates Experimental Nonalcoholic Steatohepatitis and the Progression of Thioacetamide-Induced Liver Fibrosis by Blocking the MAPK and TGF-β/Smad3 Signaling Pathways. Ann. Hepatol..

[34] Kennedy O.J., Fallowfield J.A., Poole R., Hayes P.C., Parkes J., Roderick P.J. (2021). All Coffee Types Decrease the Risk of Adverse Clinical Outcomes in Chronic Liver Disease: A UK Biobank Study. BMC Public Health.

[35] Brandt A., Nier A., Jin C.J., Baumann A., Jung F., Ribas V., García-Ruiz C., Fernández-Checa J.C., Bergheim I. (2019). Consumption of Decaffeinated Coffee Protects against the Development of Early Non-Alcoholic Steatohepatitis: Role of Intestinal Barrier Function. Redox Biol..

[36] Lu H., Tian Z., Cui Y., Liu Z., Ma X. (2020). Chlorogenic Acid: A Comprehensive Review of the Dietary Sources, Processing Effects, Bioavailability, Beneficial Properties, Mechanisms of Action, and Future Directions. Compr. Rev. Food Sci. Food Saf..

[37] Lee J., Kim J., Lee R., Lee E., Choi T.G., Lee A.S., Yoon Y.I., Park G.C., Namgoong J.M., Lee S.G. (2022). Therapeutic Strategies for Liver Diseases Based on Redox Control Systems. Biomed. Pharmacother..

[38] Loboda A., Damulewicz M., Pyza E., Jozkowicz A., Dulak J. (2016). Role of Nrf2/HO-1 System in Development, Oxidative Stress Response and Diseases: An Evolutionarily Conserved Mechanism. Cell. Mol. Life Sci..

[39] Funes S.C., Rios M., Fernández-Fierro A., Covián C., Bueno S.M., Riedel C.A., Mackern-Oberti J.P., Kalergis A.M. (2020). Naturally Derived Heme-Oxygenase 1 Inducers and Their Therapeutic Application to Immune-Mediated Diseases. Front. Immunol..

[40] Ogino N., Miyagawa K., Nagaoka K., Matsuura-Harada Y., Ogino S., Kusanaga M., Oe S., Honma Y., Harada M., Eitoku M. (2021). Role of Ho-1 against Saturated Fatty Acid-Induced Oxidative Stress in Hepatocytes. Nutrients.

[41] Boettler U., Sommerfeld K., Volz N., Pahlke G., Teller N., Somoza V., Lang R., Hofmann T., Marko D. (2011). Coffee Constituents as Modulators of Nrf2 Nuclear Translocation and ARE (EpRE)-Dependent Gene Expression. J. Nutr. Biochem..

[42] Vicente S.J.V., Ishimoto E.Y., Torres E.A.F.S. (2014). Coffee Modulates Transcription Factor Nrf2 and Highly Increases the Activity of Antioxidant Enzymes in Rats. J. Agric. Food Chem..

[43] Naveed M., Hejazi V., Abbas M., Ali A., Jilany G., Shumzaid M., Ahmad F., Babazadeh D. (2018). Chlorogenic Acid (CGA): A Pharmacological Review and Call for Further Research. Biomed. Pharmacother..

[44] Liang N., Kitts D.D. (2014). Antioxidant property of coffee components: Assessment of methods that define mechanisms of action. Molecules.

[45] Yang J.P., Shin J.H., Seo S.H., Kim S.G., Lee S.H., Shin E.H. (2018). Effects of Antioxidants in Reducing Accumulation of Fat in Hepatocyte. Int. J. Mol. Sci..

[46] Zheng X., Dai W., Chen X., Wang K., Zhang W., Liu L., Hou J. (2015). Caffeine Reduces Hepatic Lipid Accumulation through Regulation of Lipogenesis and ER Stress in Zebrafish Larvae. J. Biomed. Sci..

[47] Miao H., Ouyang H., Guo Q., Wei M., Lu B., Kai G., Ji L. (2022). Chlorogenic Acid Alleviated Liver Fibrosis in Methionine and Choline Deficient Diet-Induced Nonalcoholic Steatohepatitis in Mice and Its Mechanism. J. Nutr. Biochem..

[48] Ricchi M., Odoardi M.R., Carulli L., Anzivino C., Ballestri S., Pinetti A., Fantoni L.I., Marra F., Bertolotti M., Banni S. (2009). Differential Effect of Oleic and Palmitic Acid on Lipid Accumulation and Apoptosis in Cultured Hepatocytes. J. Gastroenterol. Hepatol..

[49] Eynaudi A., Díaz-Castro F., Bórquez J.C., Bravo-Sagua R., Parra V., Troncoso R. (2021). Differential Effects of Oleic and Palmitic Acids on Lipid Droplet-Mitochondria Interaction in the Hepatic Cell Line HepG2. Front. Nutr..

[50] Piccolis M., Bond L.M., Kampmann M., Weissman J.S., Walther T.C., Farese R.V., Piccolis M., Bond L.M., Kampmann M., Pulimeno P. (2019). Probing the Global Cellular Responses to Lipotoxicity Caused by Saturated Fatty Acids Article Probing the Global Cellular Responses to Lipotoxicity Caused by Saturated Fatty Acids. Mol. Cell.

[51] Leamy A.K., Egnatchik R.A., Young J.D. (2013). Molecular Mechanisms and the Role of Saturated Fatty Acids in the Progression of Non-Alcoholic Fatty Liver Disease. Prog. Lipid Res..

[52] Plötz T., Von Hanstein A., Krümmel B., Laporte A., Mehmeti I., Lenzen S. (2019). Structure-Toxicity Relationships of Saturated and Unsaturated Free Fatty Acids for Elucidating the Lipotoxic Effects in Human EndoC-ΒH1 Beta-Cells. Biochim. Biophys. Acta (BBA)-Mol. Basis Dis..

[53] Fujimoto T., Parton R.G. (2011). Not Just Fat: The Structure and Function of the Lipid Droplet. Cold Spring Harb. Perspect. Biol..

[54] Henne W.M., Reese M.L., Goodman J.M. (2018). The Assembly of Lipid Droplets and Their Roles in Challenged Cells. EMBO J..

[55] Petan T., Jarc E., Jusović M. (2018). Lipid Droplets in Cancer: Guardians of Fat in a Stressful World. Molecules.

[56] Zadoorian A., Du X., Yang H. (2023). Lipid droplet biogenesis and functions in health and disease. Nat. Rev. Endocrinol..

[57] Bailey A.P., Koster G., Guillermier C., Lechene C.P., Postle A.D., Gould A.P., Bailey A.P., Koster G., Guillermier C., Hirst E.M.A. (2015). Antioxidant Role for Lipid Droplets in a Stem Cell Niche of Drosophila Article Antioxidant Role for Lipid Droplets in a Stem Cell Niche of Drosophila. Cell.

[58] Miller I.P., Pavlović I., Poljšak B., Šuput D., Milisav I. (2019). Beneficial Role of ROS in Cell Survival: Moderate Increases in H2 O2 Production Induced by Hepatocyte Isolation Mediate Stress Adaptation and Enhanced Survival. Antioxidants.

[59] Chitraju C., Trötzmüller M., Hartler J., Wolinski H., Thallinger G.G., Lass A., Zechner R., Zimmermann R., Köfeler H.C., Spener F. (2012). Lipidomic Analysis of Lipid Droplets from Murine Hepatocytes Reveals Distinct Signatures for Nutritional Stress. J. Lipid Res..

[60] Herms A., Bosch M., Ariotti N., Reddy B.J.N., Fajardo A., Fernández-Vidal A., Alvarez-Guaita A., Fernández-Rojo M.A., Rentero C., Tebar F. (2013). Cell-to-Cell Heterogeneity in Lipid Droplets Suggests a Mechanism to Reduce Lipotoxicity. Curr. Biol..

[61] Magdalena A., Roglans N., Bentanachs R., Gen M., Sala-vila A., Iolanda L., Rodr J., Mar R. (2020). Effects of a Low Dose of Ca Ff Eine Alone or as Part of a Green Co Ff Ee Extract, in a Rat Dietary Model of Lean Non-Alcoholic Fatty Liver Disease without Inflammation. Nutrients.

[62] Du X., Huang Q., Guan Y., Lv M., He X., Fang C., Wang X., Sheng J. (2018). Caffeine Promotes Conversion of Palmitic Acid to Palmitoleic Acid by Inducing Expression of Fat-5 in Caenorhabditis Elegans and Scd1 in Mice. Front. Pharmacol..

[63] Aljohani A.M., Syed D.N., Ntambi J.M. (2017). Insights into Stearoyl-CoA Desaturase-1 Regulation of Systemic Metabolism. Trends Endocrinol. Metab..

[64] Dalla Valle A., Vertongen P., Spruyt D., Lechanteur J., Suain V., Gaspard N., Brion J.P., Gangji V., Rasschaert J. (2019). Induction of Stearoyl-CoA 9-Desaturase 1 Protects Human Mesenchymal Stromal Cells Against Palmitic Acid-Induced Lipotoxicity and Inflammation. Front. Endocrinol..

[65] Thörn K., Hovsepyan M., Bergsten P. (2010). Reduced Levels of SCD1 Accentuate Palmitate-Induced Stress in Insulin-Producing β-Cells. Lipids Health Dis..

[66] Yang C., Lim W., Bazer F.W., Song G. (2018). Down-Regulation of Stearoyl-CoA Desaturase-1 Increases Susceptibility to Palmitic-Acid-Induced Lipotoxicity in Human Trophoblast Cells. J. Nutr. Biochem..

[67] Dobrzyn P., Dobrzyn A., Miyazaki M., Cohen P., Asilmaz E., Hardie D.G., Friedman J.M., Ntambi J.M. (2004). Stearoyl-CoA Desaturase 1 Deficiency Increases Fatty Acid Oxidation by Activating AMP-Activated Protein Kinase in Liver. Proc. Natl. Acad. Sci. USA.

[68] Peláez R., Pariente A., Pérez-Sala Á., Larráyoz I.M. (2020). Sterculic Acid: The Mechanisms of Action beyond Stearoyl-CoA Desaturase Inhibition and Therapeutic Opportunities in Human Diseases. Cells.

[69] Schott M.B., Weller S.G., Schulze R.J., Krueger E.W., Drizyte-Miller K., Casey C.A., McNiven M.A. (2019). Lipid Droplet Size Directs Lipolysis and Lipophagy Catabolism in Hepatocytes. J. Cell Biol..

[70] Schulze R.J., Krueger E.W., Weller S.G., Johnson K.M., Casey C.A. (2020). Direct Lysosome-Based Autophagy of Lipid Droplets in Hepatocytes. Proc. Natl. Acad. Sci. USA.

[71] Schulze R.J., Dri K. (2017). Hepatic Lipophagy: New Insights Into Autophagic Catabolism of Lipid Droplets in the Liver. Hepatol. Commun..

[72] Martinez-lopez N., Singh R. (2015). Autophagy and Lipid Droplets in the Liver. Annu. Rev. Nutr..

[73] Carotti S., Aquilano K., Zalfa F., Ruggiero S., Valentini F., Zingariello M., Francesconi M., Perrone G., Alletto F., Antonelli-Incalzi R. (2020). Lipophagy Impairment Is Associated With Disease Progression in NAFLD. Front. Physiol..

[74] Sinha R.A., Farah B.L., Singh B.K., Siddique M.M., Li Y., Wu Y., Ilkayeva O.R., Gooding J., Ching J., Zhou J. (2014). Caffeine Stimulates Hepatic Lipid Metabolism by the Autophagy-Lysosomal Pathway in Mice. Hepatology.

[75] Ding W.-X. (2014). Drinking Coffee Burns Hepatic Fat by Inducing Lipophagy Coupled with Mitochondrial β-Oxidation Wen-Xing. Hepatology.

[76] Kim H.M., Kim Y., Lee E.S., Huh J.H., Chung C.H. (2018). Caffeic Acid Ameliorates Hepatic Steatosis and Reduces ER Stress in High Fat Diet À Induced Obese Mice by Regulating Autophagy. Nutrition.

[77] Daemen S., Gemmink A., Brouwers B., Meex R.C.R., Huntjens P.R., Schaart G., Moonen-Kornips E., Jörgensen J., Hoeks J., Schrauwen P. (2018). Distinct Lipid Droplet Characteristics and Distribution Unmask the Apparent Contradiction of the Athlete’s Paradox. Mol. Metab..

