# Supplementary material for: Coffee Compounds Protection Against Lipotoxicity Is Associated with Lipid Droplet Formation and Antioxidant Response in Primary Rat Hepatocytes
Source: Antioxidants (Basel). 2025 Jan 31;14(2):175. doi: 10.3390/antiox14020175 (PMC11851918; doi:10.3390/antiox14020175)

Table S1. List of primers and probes sequences

Gene	Forward 5'-3'	Reverse 5'-3'	Probe 5'-3'
18S	CGGCTACCACATCCAAGGA	CCAATTACAGGGCCTCGAAA	CGCGCAAATTACCCACTCCCGA
MnSOD	CACCGAGGAGAAGTACCACGA	GAACTTCAGTGCAGGCTGAAGA	CCTGAGTTGTAACATCTCCCTTGGCCAG
HO-1	CACAGGGTGACAGAAGAGGCTAA	CTGGTCTTTGTGTTCCTCTGTCAG	CAGCTCCTCAAACAGCTCAATGTTGAGC
SOD-1	CAGGACCTCATTTTAATCCTCACTC	GTCTCCAACATGCCTCTCTTCA	CCGCTGGACCGCCATGTTTCTT
Gpx1	GGACATCAGGAGAATGGCAAGA	CGCACTTCTCAAACAATGTAAAGTTG	TTCCCTCAAGTATGTCCGACCCGGTG
CHOP	TCCTGTCCTCAGATGAAATTGG	TCAAGAGTAGTGAAGGTTTTTGATTCT	CACCTATATCTCATCCCCAGGAAACGAAGA
GRP78	AAAGAAGGTCACCCATGCAGTT	CAATAGTGCCAGCATCCTTGT	ACTTCAATGATGCACAGCGGCAAGC

Figure S1.

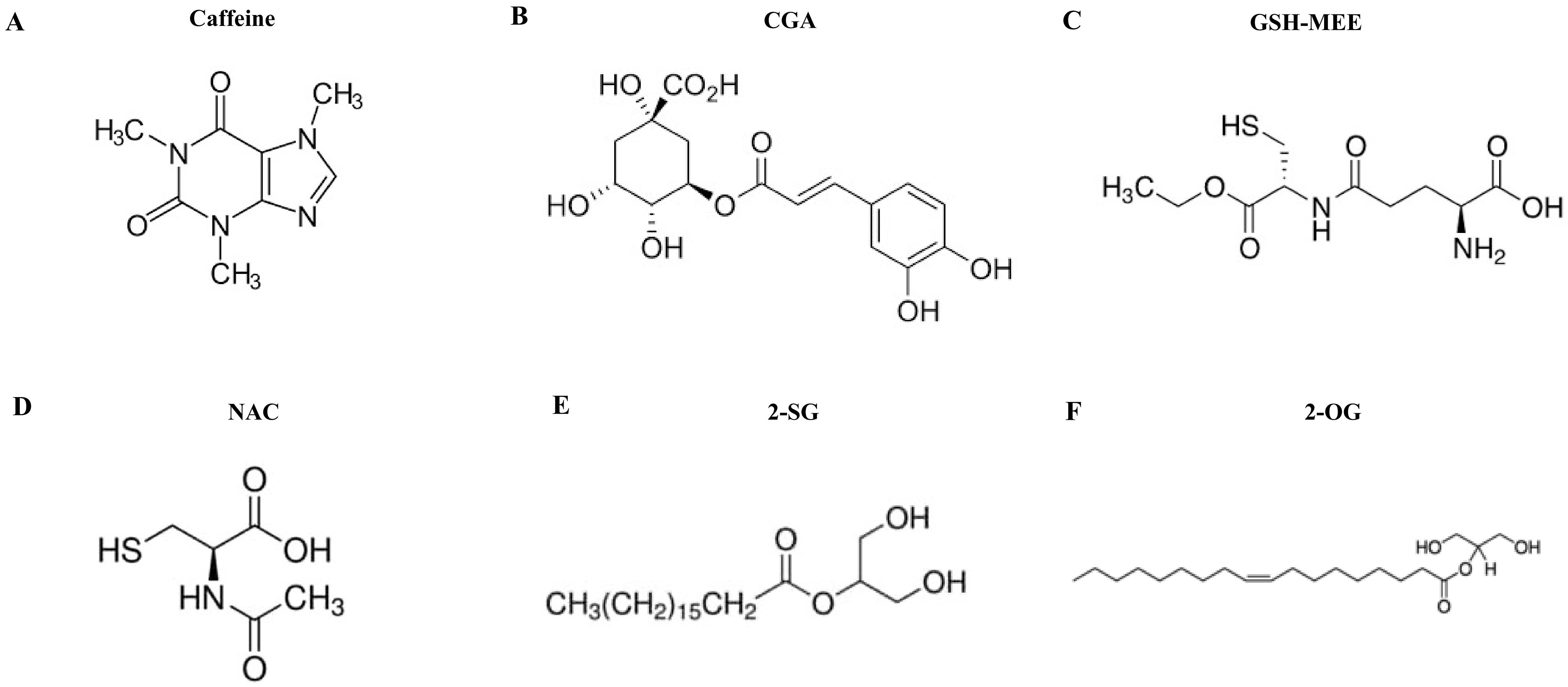

Figure S2

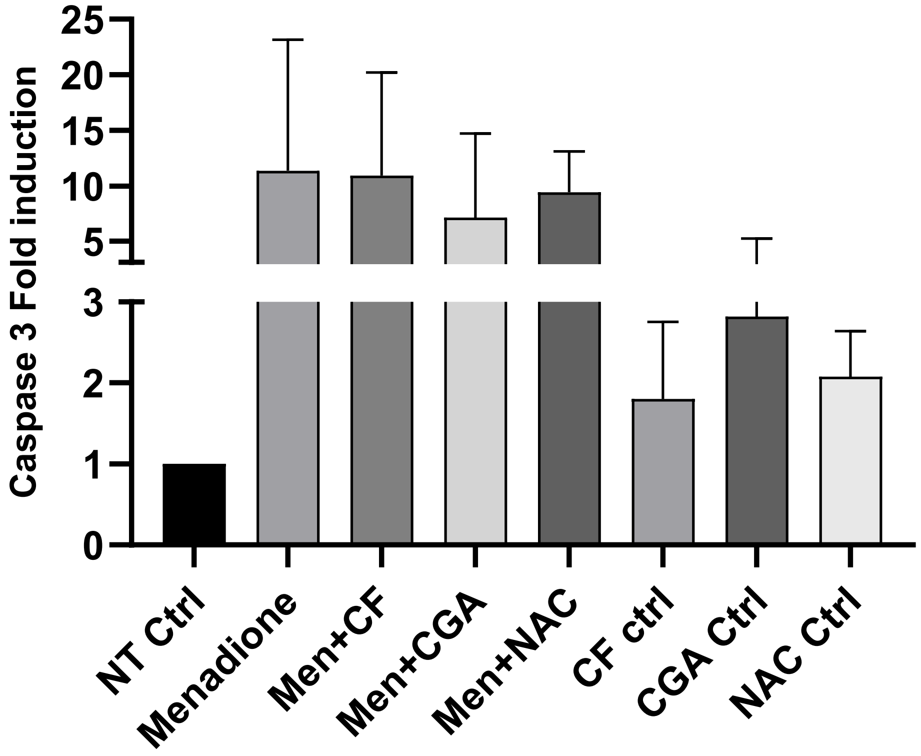

Caspase 3 Fold induction
25
20
15
10
5
3
2
1
0
NT Ctrl
Menadione
Men+CF
Men+CGA
Men+NAC
CF ctrl
CGA Ctrl
NAC Ctrl

Figure S3.

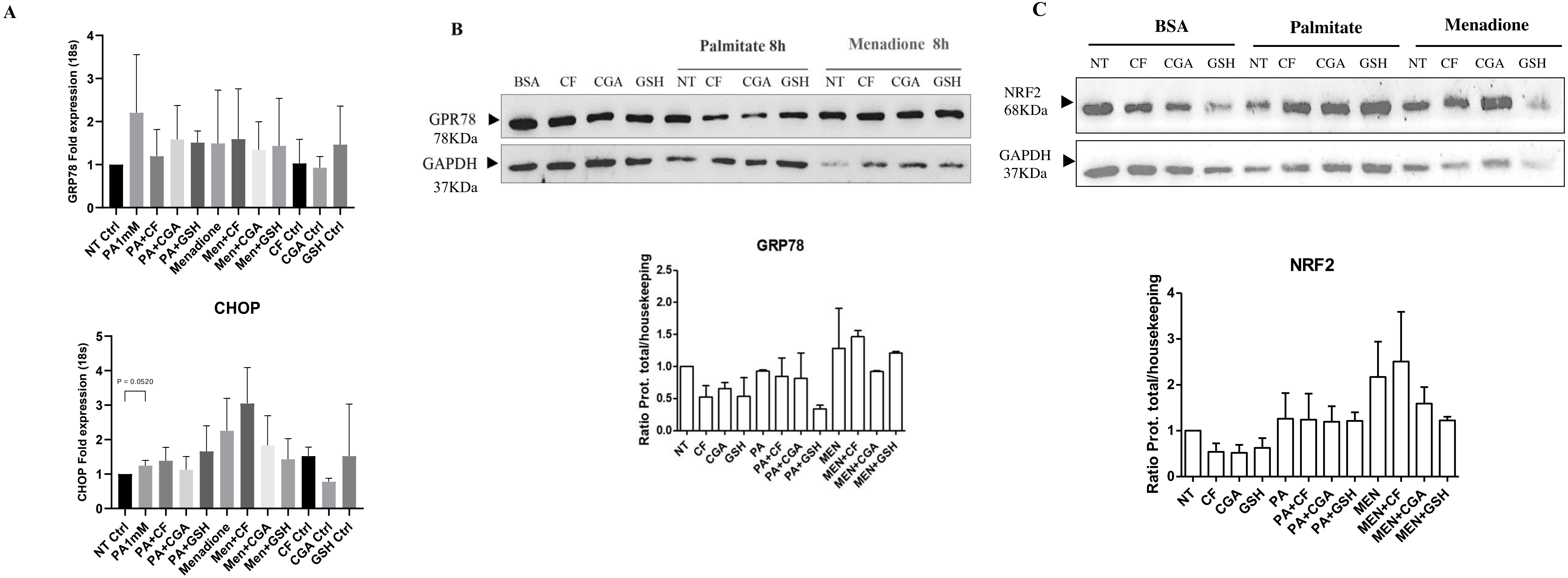
A
GRP78 Fold expression (18s)
NT Ctrl
PA1mM
PA+CF
PA+CGA
PA+GSH
Menadione
Men+CF
Men+CGA
Men+GSH
CF Ctrl
CGA Ctrl
GSH Ctrl
CHOP
CHOP Fold expression (18s)
P = 0.0520
B
Palmitate 8h
Menadione 8h
BSA CF CGA GSH NT CF CGA GSH NT CF CGA GSH
GPR78
78KDa
GAPDH
37KDa
GRP78
Ratio Prot. total/housekeeping
NT
CF
CGA
GSH
PA
PA+CF
PA+CGA
PA+GSH
MEN
MEN+CF
MEN+CGA
MEN+GSH
C
BSA
Palmitate
Menadione
NT CF CGA GSH NT CF CGA GSH NT CF CGA GSH
NRF2
68KDa
GAPDH
37KDa
NRF2
Ratio Prot. total/housekeeping

Figure S4

A

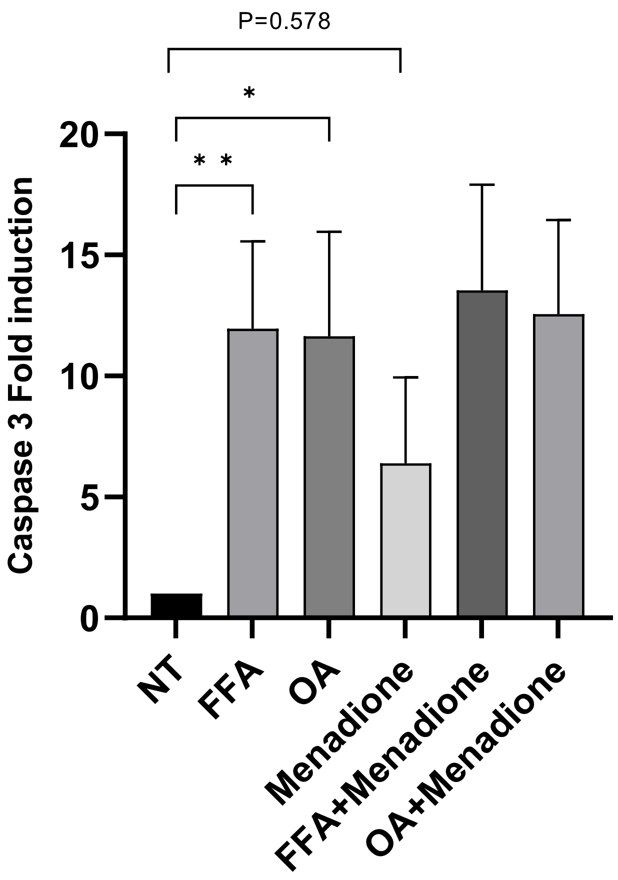

P=0.578
*
* *
20
15
10
5
0
Caspase 3 Fold induction
NT
FFA
OA
Menadione
FFA+Menadione
OA+Menadione

B

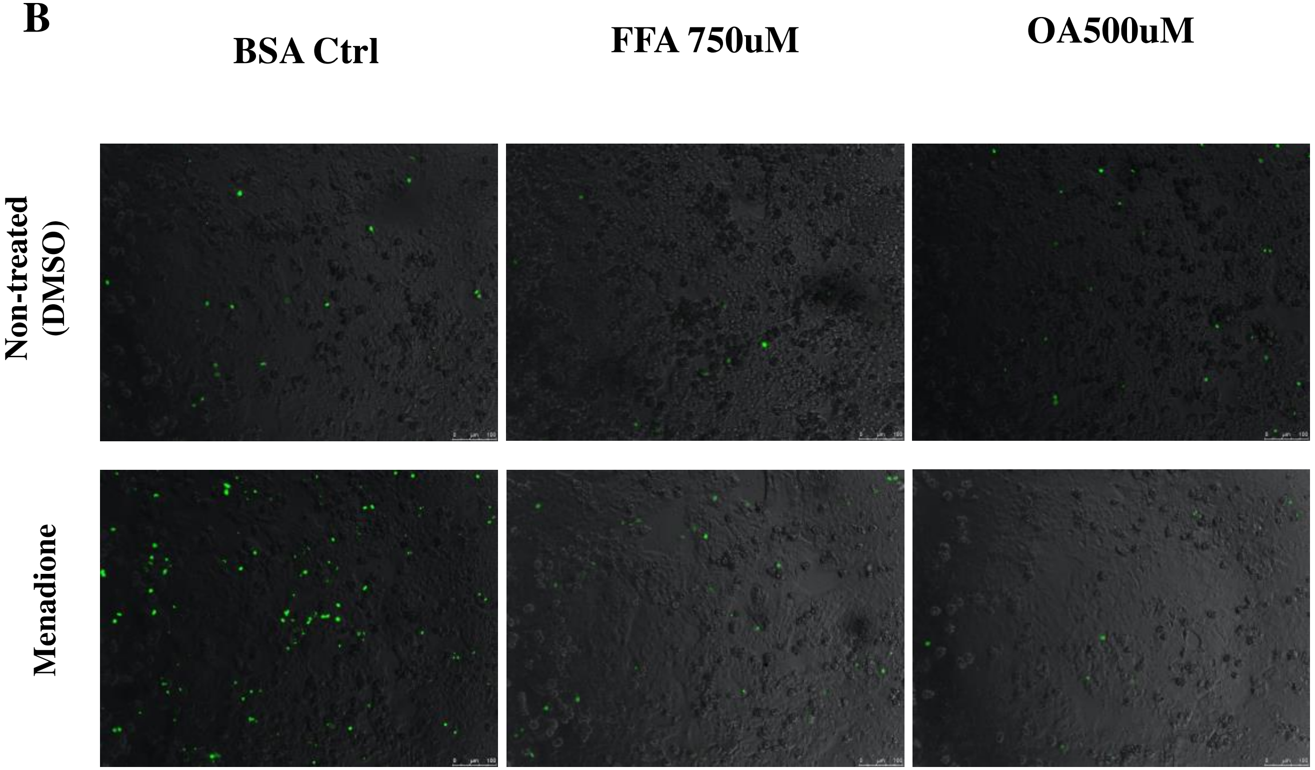

BSA Ctrl
FFA 750uM
OA500uM
Non-treated (DMSO)
Menadione

Figure S5

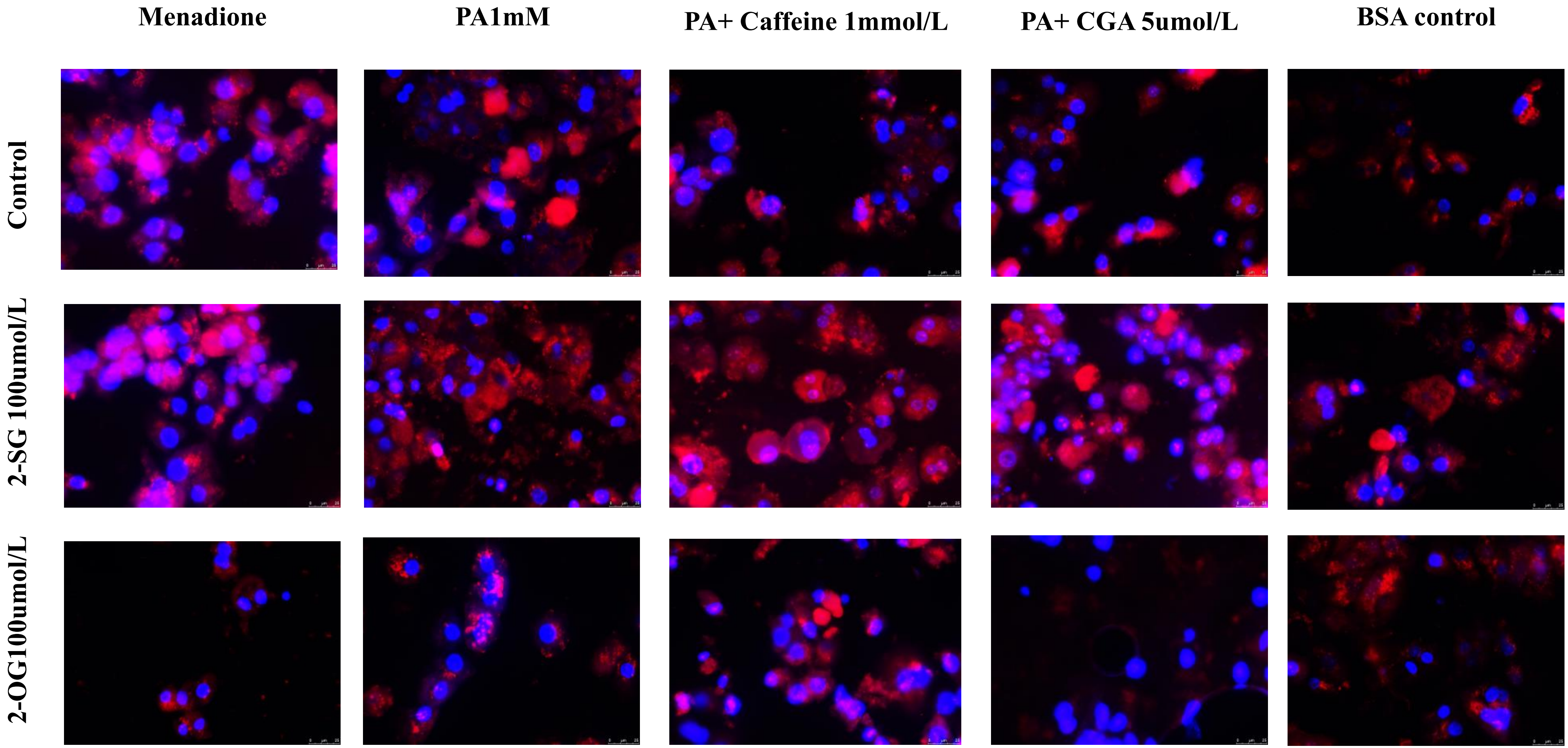
Menadione
PA1mM
PA+ Caffeine 1mmol/L
PA+ CGA 5umol/L
BSA control
Control
2-SG 100umol/L
2-OG100umol/L

Figure S6

A

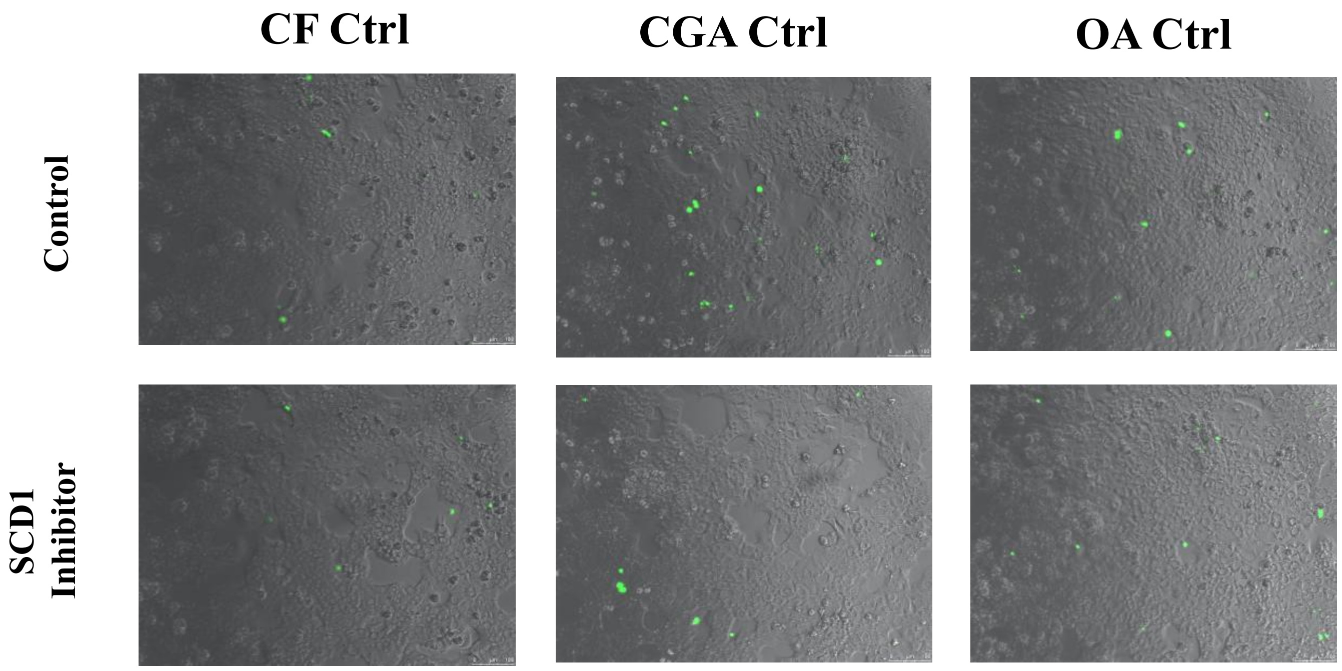

Supplement: Supplementary file 1 [file antioxidants-14-00175-s001.zip › antioxidants-3352449-supplementary.pdf]
